# METTL1‐Mediated N^7^‐Methylguanosine tRNA Modification Alleviates Cardiac Ischemia/Reperfusion Injury by Modulating Mitochondrial Energy Metabolism

**DOI:** 10.1002/mco2.70572

**Published:** 2026-01-07

**Authors:** Yue Zhang, Mingyang Leng, Ruonan Wang, Xinyuan Tang, Zhenlu Cai, Liang Wang, Xiaoqi Shao, Hongtao Diao, Qinqiang Long, Xu Li, Yingzi Wu, Yuan Jiang, Haifeng Zhang, Haihai Liang, Jiao Guo

**Affiliations:** ^1^ Key Laboratory of Glucolipid Metabolic Disorder Ministry of Education of China Guangdong Metabolic Diseases Research Center of Integrated Chinese and Western Medicine Guangdong Key Laboratory of Metabolic Disease Prevention and Treatment of Traditional Chinese Medicine Institute of Chinese Medicine Guangdong Pharmaceutical University Guangzhou China; ^2^ State Key Laboratory of Frigid Zone Cardiovascular Diseases (SKLFZCD) Department of Pharmacology (State‐Province Key Laboratories of Biomedicine‐Pharmaceutics of China Key Laboratory of Cardiovascular Research, Ministry of Education) College of Pharmacy Harbin Medical University Harbin China; ^3^ Center For Drug Research and Development Guangdong Pharmaceutical University Guangzhou China; ^4^ Key Laboratory of Tropical Biological Resources of Ministry of Education and Hainan Engineering Research Center for Drug Screening and Evaluation School of Pharmaceutical Sciences Hainan University Haikou China; ^5^ School of Life and Health Sciences Hainan University Haikou China; ^6^ Department of Cardiology Sun Yat‐sen Memorial Hospital Sun Yat‐sen University Guangzhou China

**Keywords:** myocardial ischemia/reperfusion injury, mitochondrial energy metabolism, METTL1, m^7^G tRNA modification

## Abstract

Ischemic heart disease is one of the diseases with the highest morbidity and mortality in the world. The N^7^‐methylguanosine (m^7^G) tRNA modifications are widely recognized as one of the most prevalent tRNA modifications. Nevertheless, there is still a lack of understanding regarding the roles and molecular mechanisms underlying the METTL1‐mediated m^7^G tRNA modification in cardiac ischemia/reperfusion (I/R) injury. METTL1 and m^7^G tRNA modification were upregulated in mice with I/R injury hearts and the plasma of patients with acute myocardial infarction. Thus, we constructed METTL1 knockout mice and found that silencing METTL1 alleviates I/R. Mechanistically, tRNA sequencing, MeRIP‐m^7^G‐tRNA sequencing, and Ribosome profiling sequencing were used to clarify deficiency of METTL1 reduced the levels of m^7^G tRNA modifications and m^7^G‐modified tRNAs, and consequently, downregulated the translation efficiency of ATPIF1 mRNA to restore the level of mitochondrial oxidative phosphorylation and suppress the increase of mitochondrial apoptosis. Moreover, cardiac‐specific overexpression of ATPIF1 induced myocardial hypertrophy and inhibited the protective effect of silencing METTL1 on cardiac I/R injury. Collectively, m^7^G tRNA modifications regulate the translation efficiency of ATPIF1, which eventually mediates mitochondrial energy metabolism, apoptosis, and myocardial I/R injury. The findings uncover that interfering with METTL1 and ATPIF1 represents a novel therapeutic target in myocardial I/R injury.

## Graphical abstract

During cardiac I/R injury, the level of METTL1 and tRNA m^7^G modification increases and then leads to CMs’ mitochondrial dysfunction and aggravated cardiac injury by modulating the transcription efficiency of ATPIF1.

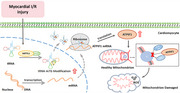

## 1 Introduction

Myocardial infarction (MI) poses a significant threat to the health of humans, causing high rates of morbidity and mortality globally [1]. The most generally used therapeutic and interventional strategies for MI patients include percutaneous coronary intervention and thrombolytic therapy [2, 3, 4]. However, patients with myocardial infarction will suffer further damage after restoring coronary blood perfusion, which is called cardiac ischemia/reperfusion (I/R) injury [5, 6]. Multiple mechanisms, including mitochondrial dysfunction, oxidative stress, and Ca^2+^ overload, contribute to I/R injury of the myocardium [7, 8, 9]. Despite important advancements in the treatment of myocardial I/R injury in recent decades, the progress in developing therapeutic strategies to mitigate I/R injury remains inadequate. Therefore, it is imperative to improve clinical outcomes by acquiring a more comprehensive comprehension of the molecular mechanism and identifying the efficient target of I/R injury.

Transfer RNAs (tRNAs) are pivotal molecules involved in decoding the genetic code and protein synthesis within cells, playing a significant part in the process of biological life [10, 11, 12]. The N^7^‐methylguanosine (m^7^G) tRNA modification, situated mainly at position 46 inside the variable loops of tRNAs, is a highly conserved modification that is vital for maintaining tRNA stability [13, 14]. Methyltransferase 1 (METTL1) and the regulatory unit WD repeat domain 4 (WDR4) form the complex that catalyzes m^7^G tRNA modification in eukaryotes [15]. The METTL1 is responsible for the catalytic process of methylation, while the ligand WDR4 plays a pivotal part in the stabilization of the methyltransferase complex [16]. Accumulating evidence has demonstrated that METTL1 is upregulated in cancers, and m^7^G tRNA modification plays a key function in the development and progression of tumors [17, 18, 19, 20, 21]. METTL1‐mediated m^7^G tRNA modification is also extensively involved in the pathogenesis of cardiac diseases [22]. For example, METTL1‐mediated m^7^G tRNA modifications have been identified as a significant contributor to myocardial fibrosis and hypertrophy [23, 24]. Nevertheless, there is still a lack of understanding regarding the roles and molecular mechanisms underlying the METTL1‐mediated m^7^G tRNA modification in cardiac I/R injury.

Mitochondrial dysfunction plays a crucial role in myocardial I/R injury [25, 26, 27]. ATPase inhibitory factor 1 (ATPIF1), a highly conserved protein of the mitochondrion, suppresses the activity of F1‐F0 ATP synthase and regulates multiple metabolic parameters, including mitochondrial cristae density, ATP synthase dimerization, aerobic glycolysis, etc [28, 29, 30]. ATPIF1 is present near the α and β catalytic subunits of F1 ATPase in the mitochondrial respiratory chain and exerts its inhibitory activity by binding to the catalytic interface of F1 ATPase [31, 32]. There is an ongoing debate regarding the role of ATPIF1 in cardiovascular disease. This apparent contradictory effect is puzzling and needs further research.

In the current investigation, we systematically explored the molecular mechanism by which METTL1‐mediated m^7^G modifications regulate ATPIF1 and its influence in I/R. We noticed that METTL1 influenced the translation efficiency of the ATPIF1 mRNA via mediating m^7^G tRNA modification, ultimately regulating cardiomyocyte mitochondrial energy metabolism, apoptosis, and I/R injury. Our findings unveil the intricate role of m^7^G tRNA methylation in I/R injury, suggesting that METTL1 and ATPIF1 could serve as potential therapeutic biotargets for myocardial I/R injury.

## 2 Results

### 2.1 Upregulation of METTL1‐Mediated M^7^G tRNA Modification in Myocardial I/R Injury

**FIGURE 1:**
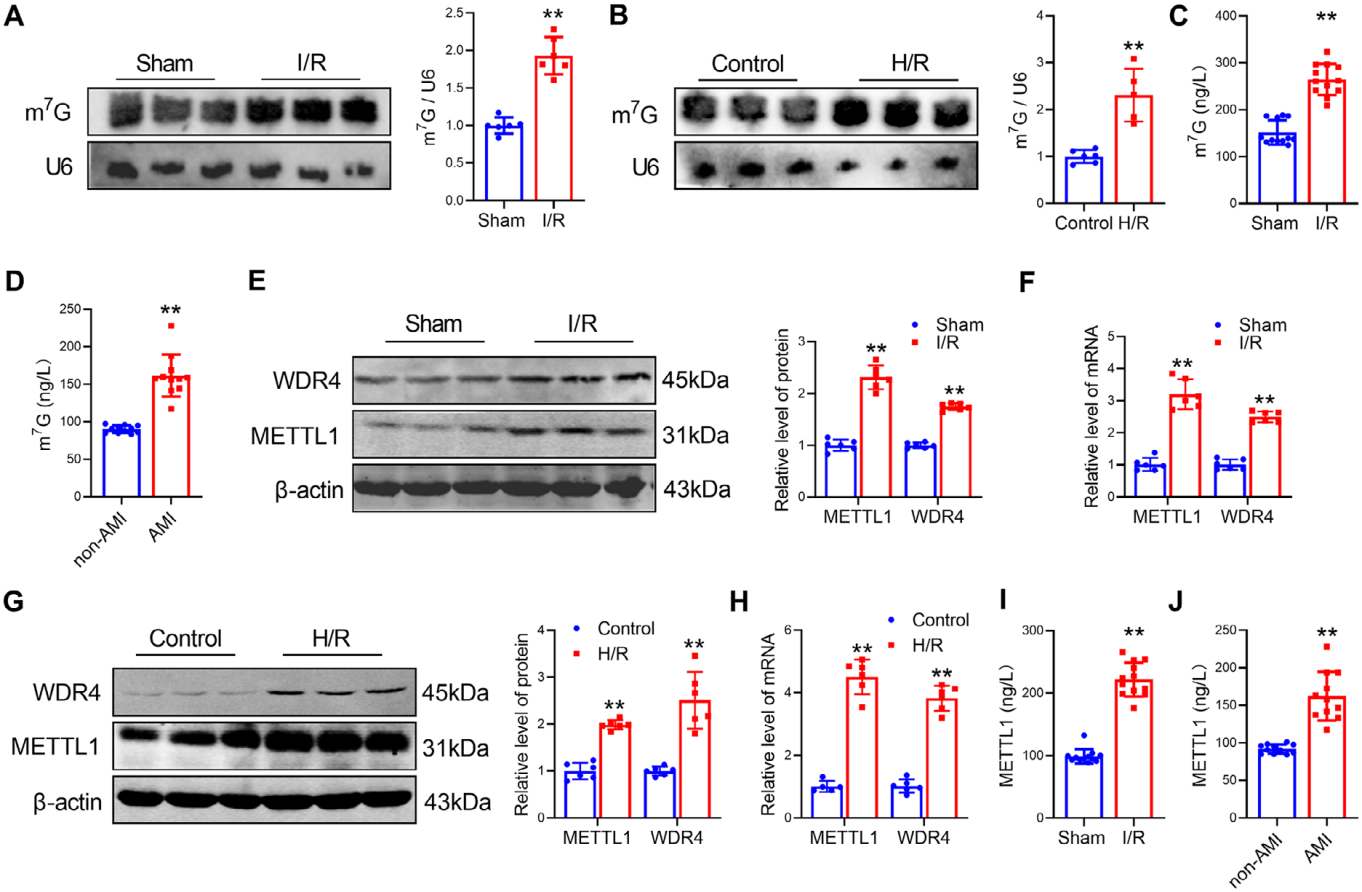
METTL1/WDR4 and m^7^G tRNA modification were upregulated in myocardial I/R injury. (A) The level of m^7^G modification in heart tissues subjected to I/R by northwestern blot. U6 snRNA was used as a loading control. *n* = 6. (B) The level of m^7^G in CMs after H/R treatment by northwestern blot. *n* = 5. (C) The m^7^G level in the plasma of mice with I/R. *n* = 12. (D) The m^7^G level in the plasma of AMI patients. *n* = 11. (E) The protein levels of METTL1 and WDR4 in cardiac tissues subjected to I/R by Western blot. *n* = 6. (F) The mRNA levels of METTL1 and WDR4 in cardiac tissues subjected to I/R by qRT‐PCR. *n* = 6. (G) The protein levels of METTL1 and WDR4 in CMs after H/R treatment. *n* = 6. (H) The mRNA levels of METTL1 and WDR4 in CMs subjected to H/R. *n* = 5. (I) The METTL1 level in the plasma of mice with I/R. n = 12. (J) The METTL1 level in the plasma of AMI patients. *n* = 11. Values represent the mean ± SD. ^*^ *p* < 0.05, ^**^ *p* < 0.01.

To investigate the contribution of m^7^G tRNA modification on cardiac I/R injury, we focused on the changes in the level of m^7^G tRNA modification upon I/R injury. First, we constructed the mouse model of I/R (Figure S1A–D). Northern blot analysis revealed a 1.9‐fold increase in the level of m^7^G tRNA modification in hearts with I/R compared with the Sham (Figure 1A). The level of m^7^G tRNA modification was also enhanced in mouse cardiomyocytes (CMs) undergoing hypoxia/reoxygenation (H/R) (Figure 1B). Especially, we observed that the level of m^7^G tRNA modifications was dramatically upregulated in the plasma of mice with I/R and in the plasma of AMI patients (Figure 1C,D).

The m^7^G modification is adjusted by the METTL1/WDR4 complex; we measured the levels of both METTL1 and WDR4 in I/R injury. The levels of protein and mRNA expression of METTL1/WDR4 were remarkably upregulated in I/R injury hearts (Figure 1E,F). Similar to the findings observed in vivo results, the levels of METTL1/WDR4 expression were also obviously raised in cardiomyocytes induced by H/R stimulation (Figure 1G,H). Moreover, immunohistochemistry (IHC) staining also showed the elevated METTL1 expression in hearts treated with I/R (Figure S1D). Consistently, METTL1 was elevated in the plasma of AMI patients and mice (Figure 1I,J). Taken together, these data underscore the necessary function of m^7^G tRNA modification and METTL1/WDR4 in cardiac I/R injury.

### 2.2 Inhibition of METTL1 Improves Cardiac I/R Injury

**FIGURE 2:**
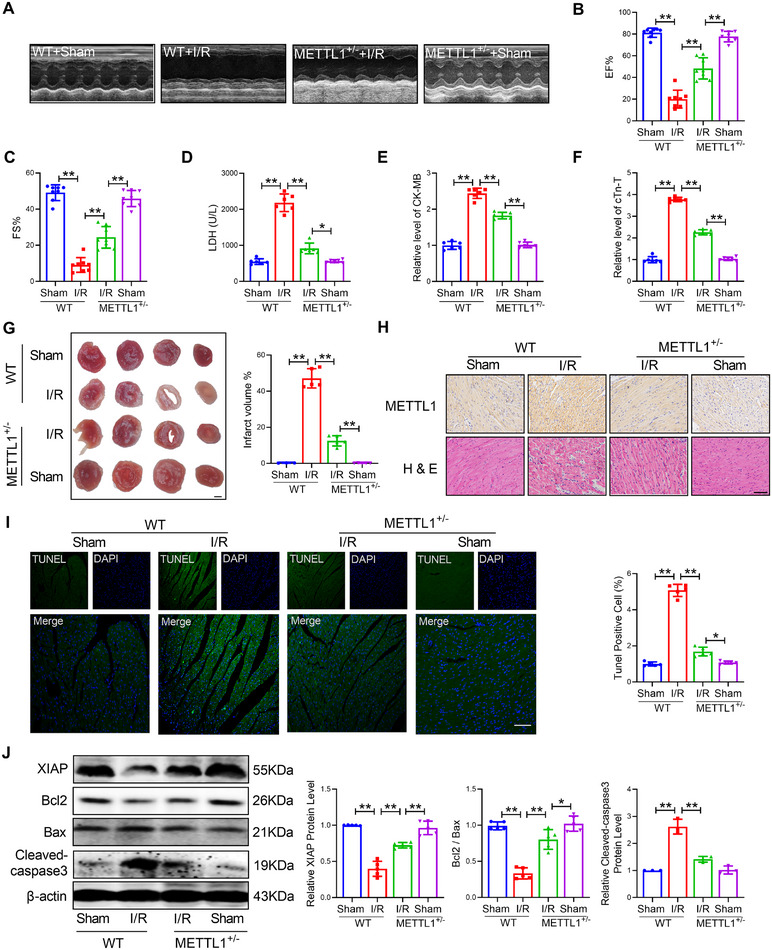
Silencing the expression of METTL1 alleviates cardiac I/R injury. (A–C) Electrocardiography was used to assess the cardiac function of wild‐type (WT) and METTL1^+/−^ mice upon I/R injury. *n* = 8. (D) The level of LDH in serum. *n* = 6. (E) The level of CK‐MB in serum. *n* = 6. (F) The level of cTn‐T in serum. *n* = 6. (G) Infarct size of I/R hearts by 2,3,5‐triphenyltetrazolium chloride (TTC) staining. Scale bar, 2 mm. *n* = 3. (H) H&E, and immunohistochemical detection of WT and METTL1^+/−^ mice with I/R injury. Scale bars: 50 µm. *n* = 5. (I) TUNEL staining was used to identify cardiomyocyte apoptosis. Scale bar: 20 µm. *n* = 5. (J) The protein levels of XIAP, Bcl2, Bax, and cleaved‐caspase3. XIAP, Bcl2, and Bax. *n* = 5. Cleaved‐caspase3. *n* = 3. Values represent the mean ± SD. ^*^ *p* < 0.05, ^**^ *p* < 0.01.

To further explore the critical function of METTL1 in myocardial injury, we utilized CRISPR/Cas9 techniques to engineer an METTL1 knockout (METTL1^+/−^) mouse model. Subsequently, we induced myocardial I/R injury through surgery and examined the level of METTL1. Successful knockout of METTL1 was confirmed by Western blot and qRT‐PCR (Figure S2A,B). The echocardiographic examination suggested that METTL1^+/−^ mice prominently improved cardiac function, as manifested by restoration of ejection fraction (EF) and fractional shortening (FS) compared with age‐matched myocardial I/R injury mice (Figure 2A–C). Lactate dehydrogenase (LDH) is an enzyme that exists in the cytoplasm of cardiomyocytes and is released into the serum when cardiomyocytes are damaged. After myocardial I/R injury, there was an obvious elevation of LDH levels, and the change was mitigated by the knockout of METTL1 (Figure 2D). The myocardial injury markers creatine kinase MB isoenzyme (CK‐MB) and cardiac troponin T (cTn‐T) also show similar changes (Figure 2E,F). Consistently, knockout of METTL1 not only significantly decreased the infarct size under cardiac I/R injury, but also improved myocardial structure and reduced inflammatory cell infiltration (Figure 2G,H). Furthermore, knocking out the expression of METTL1 decreased the number of TUNEL (TdT‐mediated dUTP nick end labeling)—positive cells (Figure 2I). Compared with I/R injury mice, knockout of METTL1 inhibited the level of Bax (Bcl2‐associated X) and cleaved‐caspase3, whilst promoting the level of Bcl2 (B‐cell lymphoma 2) and XIAP (X‐linked inhibitor of apoptosis protein), then downregulated the ratio of Bcl2/Bax (Figure 2J).

We subsequently assessed the impact of METTL1 on CM injury in vitro. The transfection of METTL1‐siRNA significantly decreased METTL1 expression in CM (Figure S2C,D). The silence of METTL1 resulted in downregulation of the level of Bax and cleaved‐caspase3, upregulation of the level of Bcl2 and XIAP, and reduced TUNEL‐positive cells (Figure S2E,F). Taken together, both in vivo and in vitro research suggest that suppression of METTL1 exerts protective effects during cardiac injury.

### 2.3 METTL1 Deletion Regulates Cardiac Injury via Improving Mitochondrial Dysfunction

**FIGURE 3:**
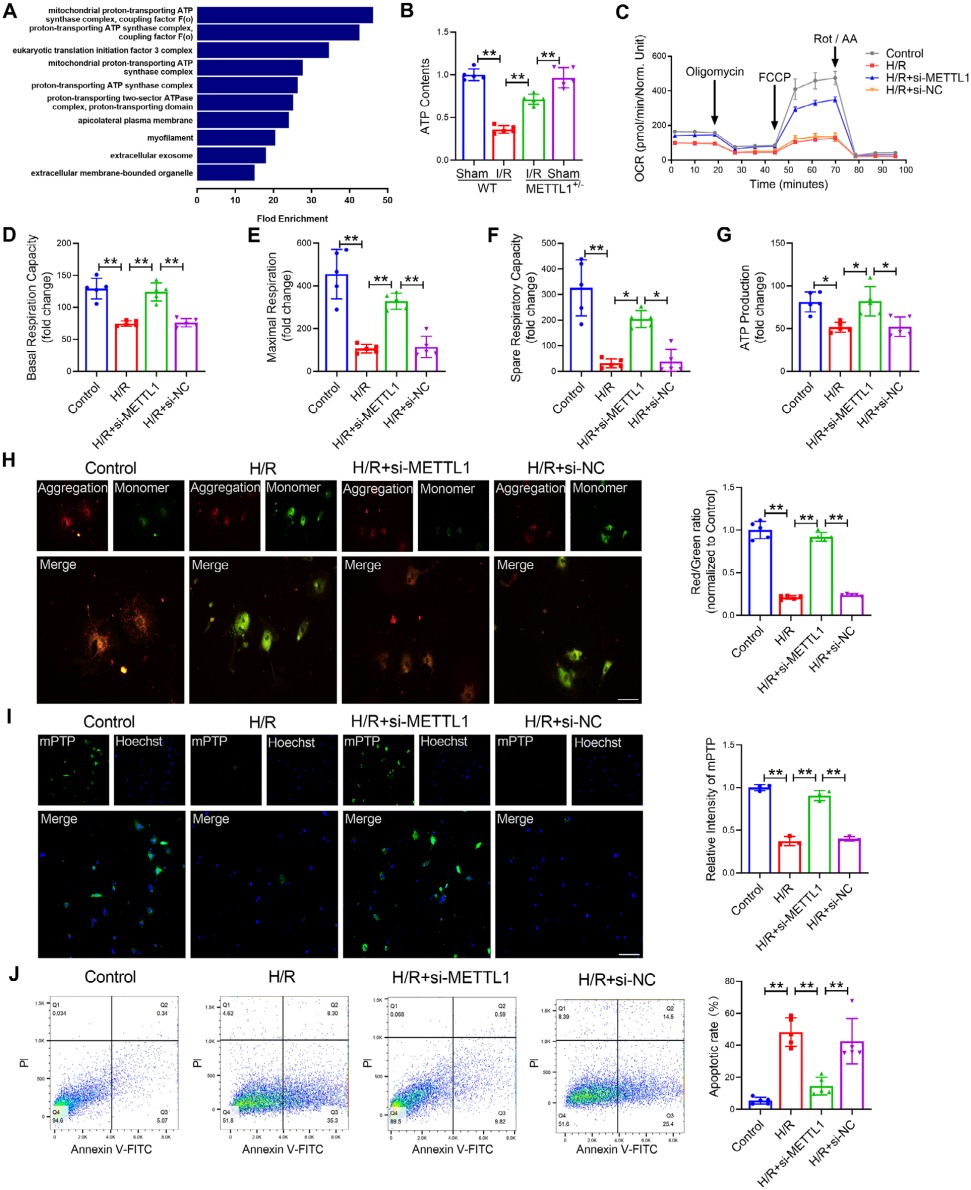
Silencing the expression of METTL1 inhibits mitochondrial dysfunction. (A) Gene ontology analysis using the differentially expressed mRNA. (B) Effects of METTL1 knockdown on ATP content in mice. *n* = 5. (C) Quantitative statistical analysis of OCR in CMs treated with H/R after transfection with METTL1‐siRNA. *n* = 5. (D) Quantitative statistical analysis of basal respiration in CMs treated with H/R after transfection with METTL1‐siRNA. *n* = 5. (E) Quantitative statistical analysis of maximal respiration in CMs treated with H/R after transfection with METTL1‐siRNA. *n* = 5. (F) Quantitative statistical analysis of spare respiratory capacity in CMs treated with H/R after transfection with METTL1‐siRNA. *n* = 5. (G) Quantitative statistical analysis of ATP production in CMs treated with H/R after transfection with METTL1‐siRNA. *n* = 5. (H) JC‐1 staining in CMs was used to detect mitochondrial membrane potential. Scale bar: 50 µm. *n* = 5. (I) mPTP staining in CMs was performed to detect mitochondrial permeability. Scale bar: 50 µm. *n* = 3. (J) Flow cytometry detection of cell apoptosis. *n* = 3. Values represent the mean ± SD. ^*^ *p* < 0.05, ^**^ *p* < 0.01.

Subsequently, to further elucidate the molecular mechanism of METTL1 in cardiac I/R injury, we analyzed the Ribosome profiling sequencing of H/R stimulation primary cardiomyocytes treated with METTL1‐siRNA or NC‐siRNA. Gene ontology analysis suggests that differentially translation efficiency downregulated (TE‐down) mRNAs are obviously enriched in mitochondrial and ATP‐related pathways (Figure 3A). So, we first performed an ATP assay to determine the function of METTL1 in regulating cardiac mitochondrial energy metabolism. We found the ATP content significantly decreased in I/R heart and H/R cardiomyocyte, but knockout of METTL1 significantly restored ATP production (Figure 3B; Figure S3A). Next, we examined the oxygen consumption rate (OCR) and basal respiration in CMs with H/R stimulation. The results demonstrated a significant reduction in OCR and basal respiration after H/R stimulation, suggesting decreased cellular energy requirements in the basal state. Importantly, these effects were reversed following the silencing of METTL1 (Figure 3C,D). Furthermore, in H/R‐cultured CMs, there was a notable decrease in maximal respiration, ATP production, and spare respiratory capacity, indicating a reduced capacity to meet energy demands. However, in METTL1‐silenced cardiomyocytes, these reductions were significantly reversed (Figure 3E–G). These data indicated that silence of METTL1 improved cardiac mitochondrial energy metabolism.

We next determined the function of METTL1 in regulating cardiac mitochondrial integrity and function after silencing METTL1 through JC‐1 staining, MitoSOX staining, and mitochondrial permeability transition pore (mPTP) staining. We observed that the function and structure of myocardial mitochondria were damaged under H/R injury. An index of mitochondrial bioenergetics, mitochondrial membrane potential, was destroyed by H/R injury but restored by knockout of METTL1 (Figure 3H). In addition, mitochondrial ROS, as the byproduct of the metabolism of mitochondria, was notably added in CMs with H/R stimulation but was reversed by deletion of METTL1 (Figure S3B). Similarly, after knockout of METTL1, CMs exhibit a reduction in the opening of mPTP (Figure 3I). The level of apoptosis measured by flow cytometry also suggests that MPTP opening leads to apoptosis rather than necrosis (Figure 3J). On the other hand, in I/R heart or H/R CM, the proteins of OPA1 (optic atrophy 1) and Mfn2 (Mitofusin 2) were downregulated, and the proteins of Drp1 (dynamin related protein 1) and Fis1 (mitochondrial fission 1) were upregulated, but they were maintained at normal levels during knockout of METTL1 (Figure S3C,D). Knockout of METTL1 inhibited mitochondrial fission and promoted mitochondrial fusion. Importantly, upon I/R injury, the leakage level of cytochrome and the protein levels of cleaved‐caspase9 were decreased in METTL1^+/−^ mice than in WT mice, suggesting silence of METTL1 had the role of antimitochondrial apoptosis (Figure S3E,F). The outcomes obtained from both in vivo and in vitro strongly display that METTL1 takes a pivotal role in the regulation of cardiac injury by ameliorating mitochondrial energy metabolism and inhibiting the level of mitochondrial ROS and apoptosis.

### 2.4 METTL1 Regulates M^7^G tRNA Modification, tRNA Expression, and Affects Global mRNA Translation in Cardiac I/R Injury

**FIGURE 4:**
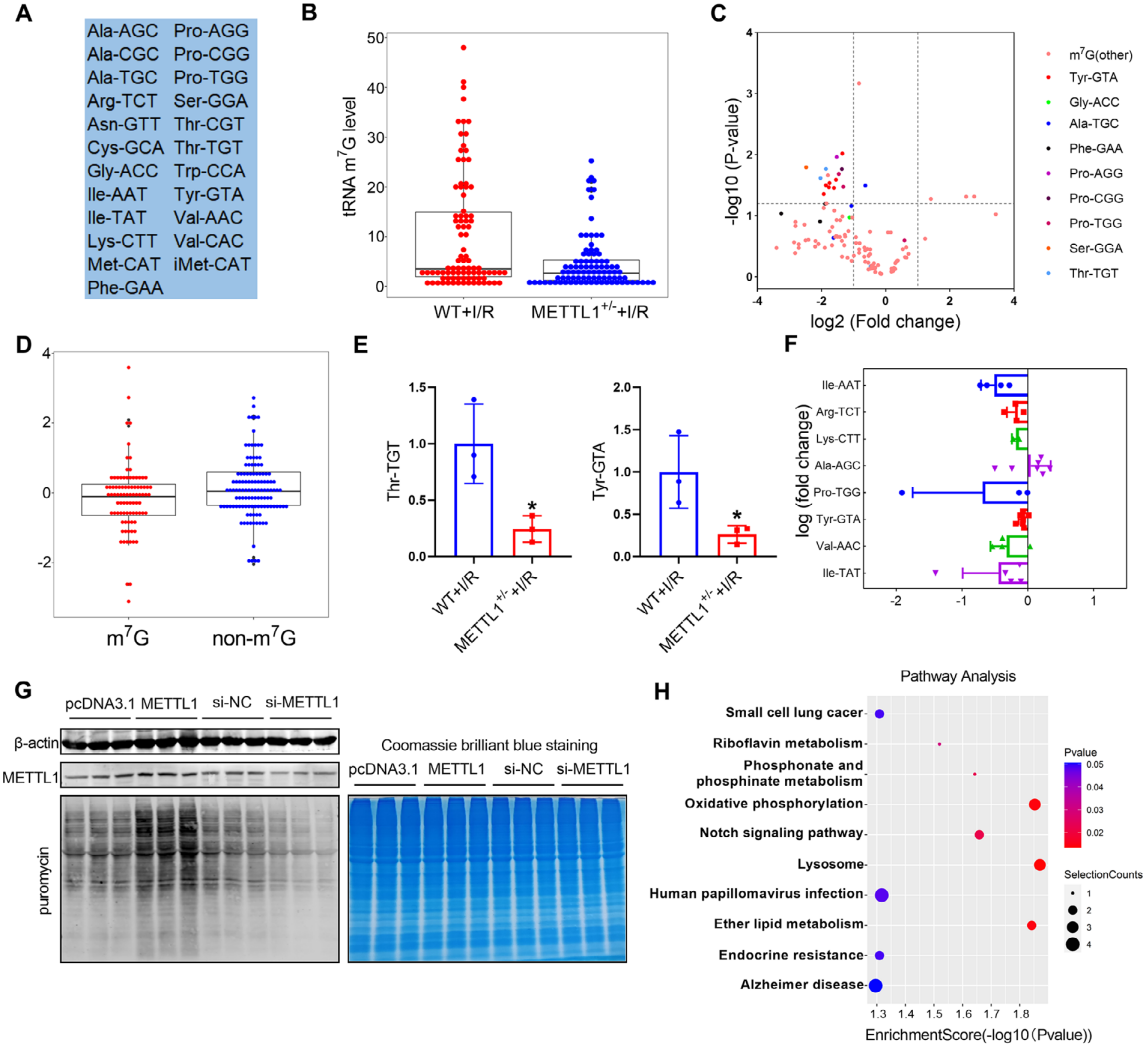
METTL1 regulates m^7^G tRNA modification, tRNA expression, and mRNA translation. (A) List of m^7^G‐modified tRNAs identified in CMs. (B) Quantification of m^7^G level on m^7^G‐modified tRNAs. (C) Changes in m^7^G tRNA modification upon knockout of METTL. (D) The expression levels of m^7^G‐modified tRNAs and non‐m^7^G‐modified tRNAs. (E) Expression profile of m^7^G modification in the METTL1 knockdown and WT mice with I/R injury by MeRIP‐tRNA‐seq. (F) Changes in tRNA abundance upon METTL1 knockdown. (G) Puromycin intake assay of CMs with METTL1‐siRNA or METTL1‐pcDNA3.1. *n* = 5. (H) Pathway analysis using the TE‐down genes by KEGG. Values represent the mean ± SD. ^*^ *p* < 0.05, ^**^ *p* < 0.01.

To determine how METTL1 ameliorates mitochondrial dysfunction, we performed tRNA‐seq and MeRIP‐m^7^G‐tRNA‐seq to profile tRNA expression and m^7^G tRNA modification. In CMs, a total of twenty‐three tRNAs that contain m^7^G modification were identified (Figure 4A). The sequencing results suggest that knockout of METTL1 significantly reduces the m^7^G signal in m^7^G‐modified tRNAs (Figure 4B,C). Northwestern blot also confirmed the reduction of m^7^G modification levels in METTL1 knockout mice (Figure S4A). Furthermore, we analyzed the expression of tRNAs and found that knockout of METTL1 resulted in reduced expression levels of most of the m^7^G‐modified tRNAs, but had few effects on the expression of non‐m^7^G‐modified tRNAs (Figure 4D). Our anti‐m^7^G tRNA‐seq and MeRIP‐seq data confirmed that the levels of m^7^G‐modified tRNAs and m^7^G tRNA modification were downregulated after METTL1 knockout (Figure 4E,F; Figure S4B). Overall, through the tRNA m^7^G methyltransferase activity, METTL1 affects both tRNA modification and expression.

In view of the tRNA‐mediated mRNA translation process, we next examined whether METTL1 affects mRNA translation in CMs. The puromycin intake assay showed that overexpression of METTL1 increased puromycin levels, indicating increased overall mRNA translation in METTL1‐overexpression cells (Figure 4G; Figure S4C). The knockdown of METTL1 decreased puromycin levels, indicating reduced overall mRNA translation in METTL1‐knockdown cells (Figure 4G; Figure S4C). These findings revealed that the expression of tRNA and the translation of mRNA in CMs depend on METTL1‐mediated m^7^G tRNA modification. Next, we further analyzed the results of the Ribosome profiling sequencing. KEGG analysis showed TE‐down genes were markedly enriched in the oxidative phosphorylation pathway (Figure 4H).

### 2.5 METTL1 Regulates Cardiac Injury Through ATPIF1

**FIGURE 5:**
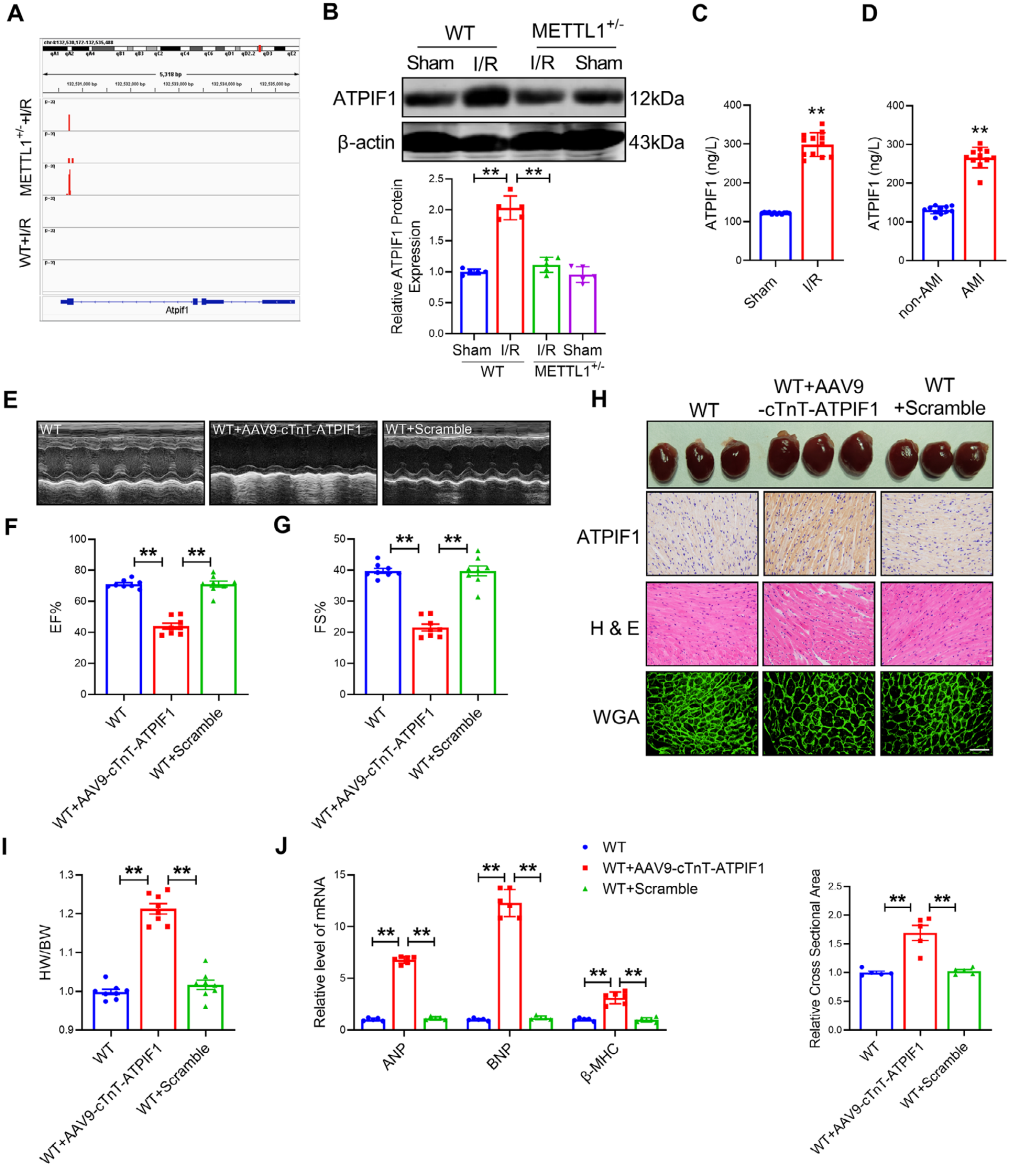
ATPIF1 is a key target of METTL1 and induces myocardial hypertrophy. (A) Ribosome profiling sequencing analyzed ATPIF1 expression. (B) The protein level of ATPIF1 by Western blot. *n* = 5. (C) Level of ATPIF1 in the plasma of mice with I/R. *n* = 12. (D) Level of ATPIF1 in the plasma of patients with AMI. *n* = 11. (E‐G) Cardiac function of WT and overexpression of ATPIF1 mice by echocardiograph. n = 8. (H) H&E, immunohistochemical detection of ATPIF1 and WGA of mice heart. Scale bars: 50 µm. *n* = 5. (I) Comparisons of the HW/BW of mice treated with AAV9‐cTnT‐ATPIF1. *n* = 8. (J) qRT‐PCR analyzed the mRNA expression of ANP, BNP, and β‐MHC in mice treated with AAV9‐cTnT‐ATPIF1. *n* = 5. Values represent the mean ± SD. ^*^ *p* < 0.05, ^**^ *p* < 0.01.

Next, we further analyzed the gene with TE‐down in mitochondria, ATP, and oxidative phosphorylation pathways and revealed that the level of protein translation efficiency with ATPIF1, SLC25A3, and ATP5G3 was significantly reduced (Figure 5A). Their expressions of protein were downregulated in METTL1^+/−^ mice hearts (Figure 5B; Figure S5A). Surprisingly, PCR assays revealed significant alterations in the mRNA levels of SLC25A3 and ATP5G3 following METTL1 knockout, which suggests the possibility of additional pathways mediating the observed phenomenon, not solely through m^7^G tRNA modification (Figure S5B). Notably, the I/R injury induced an increase in the mRNA levels of ATPIF1; the mRNA levels of ATPIF1 did not show a significant change upon knockout of METTL1, which is different from the changes in protein levels of ATPIF1 (Figure S5B,C). This prompts the consideration that ATPIF1 may serve as a pivotal target in METTL1‐mediated mitochondrial damage and myocardial injury. To further explore the role of ATPIF1 in cardiac ischemia/reperfusion injury, we investigated the protein levels of ATPIF1 in H/R injury CMs and I/R injury hearts, revealing an upregulation of ATPIF1 in cardiac injury (Figure S5D,E). Furthermore, ATPIF1 was also upregulated in the plasma of patients with AMI and in the plasma of mice with I/R (Figure 5C,D). In summary, these data indicated that METTL1‐mediated m^7^G tRNA modification exerted a significant role in maintaining mitochondrial homeostasis and energy metabolism by regulating ATPIF1 mRNA translation.

### 2.6 Overexpression of ATPIF1 Induces Myocardial Hypertrophy

To fully understand the role of ATPIF1, we constructed an adeno‐associated virus serotype 9 (AAV9) vector carrying ATPIF1 to overexpression of ATPIF1 in mice hearts. The research results showed that the overexpression of ATPIF1 impaired cardiac function and induced myocardial hypertrophy at eight weeks (Figure 5E,H). Furthermore, overexpression of ATPIF1 elevated the ratio of heart weight (HW) / body weight (BW) and the mRNA levels of β‐myosin heavy chain (β‐MHC), brain natriuretic peptide (BNP), and atrial natriuretic peptide (ANP) (Figure 5I,J). These data implied that overexpression of ATPIF1 induces myocardial hypertrophy.

### 2.7 Knockdown of ATPIF1 Alleviates Cardiac I/R Injury

**FIGURE 6:**
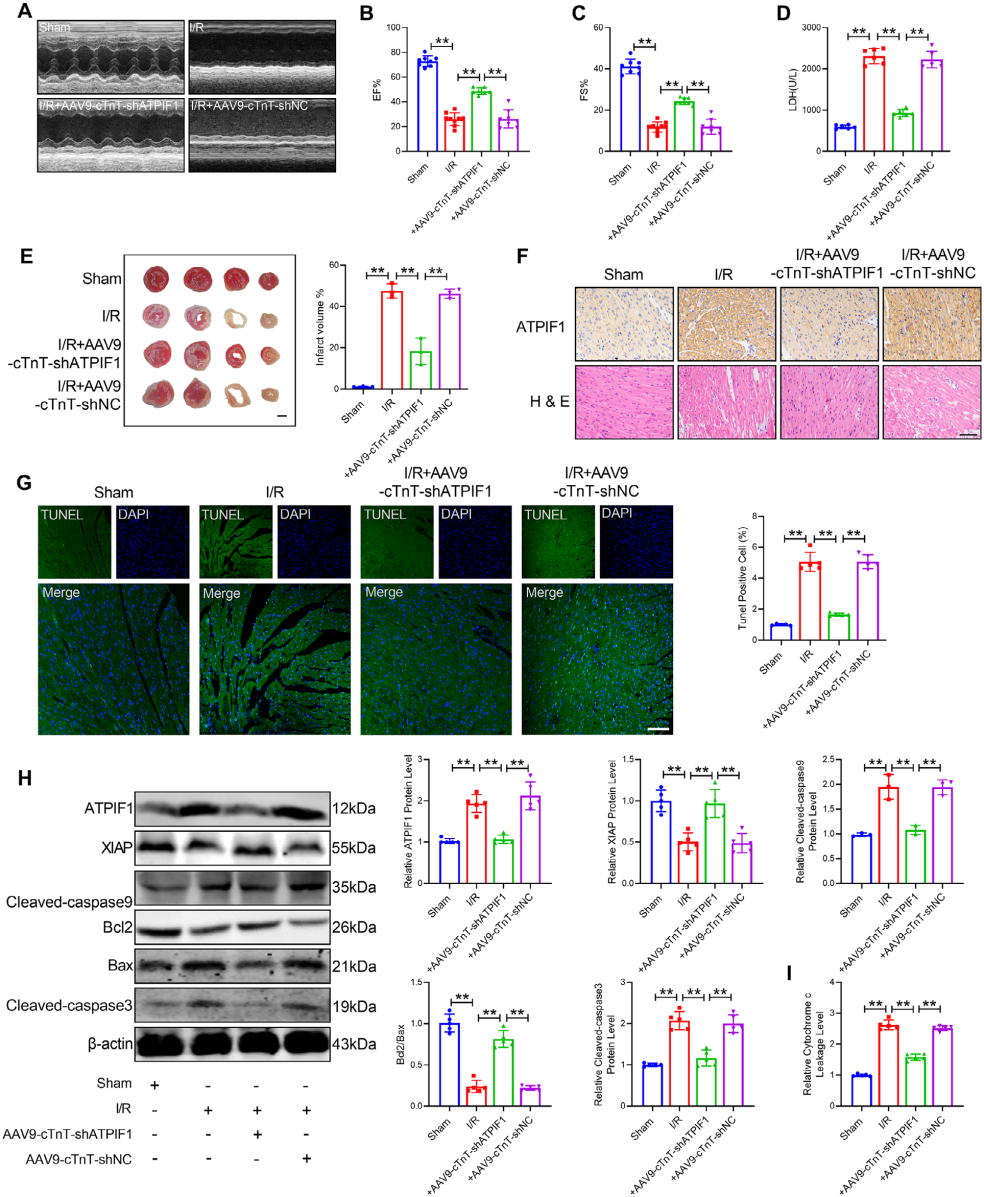
ATPIF1 improved cardiac I/R injury. (A–C) Cardiac function of WT and ATPIF1 knockdown mice with I/R injury by echocardiograph. *n* = 8. (D) Level of LDH in serum. *n* = 6. (E) Infarct size of I/R hearts by TTC staining. Scale bar: 2 mm. *n* = 3. (F) H&E and IHC staining of mice left ventricles. Scale bars, 50 µm. *n* = 5. (G) TUNEL staining was used to identify cardiomyocyte apoptosis in the border zone. Scale bar: 20 µm. *n* = 5. (H) The protein levels of Bcl2, Bax, ATPIF1, XIAP, cleaved‐caspase9, and cleaved‐caspase3. Bcl2, Bax, ATPIF1, XIAP, and cleaved‐caspase3. *n* = 5. Cleaved‐caspase9. *n* = 3. (I) The leakage level of cytochrome c. *n* = 5. Values represent the mean ± SD. ^*^ *p* < 0.05, ^**^ *p* < 0.01.

To identify the role of ATPIF1 in myocardial injury, we also constructed an AAV9 vector carrying ATPIF1‐shRNA to knock down ATPIF1. Successfully knocking down ATPIF1 was proved by PCR with cardiac tissue of knockdown of ATPIF1 mice (Figure S6A). Echocardiography results exhibited that the cardiac function of ATPIF1 knockdown mice was obviously improved (Figure 6A–C). Consistently, knocking down ATPIF1 reversed the increase in the levels of LDH, CK‐MB, cTn‐T, infarct size, and inflammatory cell infiltration caused by I/R (Figure 6D–F; Figure S6B,C). Moreover, I/R‐elicited TUNEL‐positive cells upregulation was inhibited by ATPIF1 knockdown in heart tissue (Figure 6G). We also evaluated the influence of ATPIF1 knockdown on H/R‐induced cardiomyocyte injury (Figure S6D). Upon H/R injury, the mitochondrial membrane potential was increased in ATPIF1 knockdown CMs than control (Figure S6E). H/R stimulation led to open mPTP in CMs, which was significantly improved with ATPIF1 knockdown (Figure S6F). Furthermore, ATPIF1 knockdown markedly decreased mitochondrial superoxide release under H/R treatment (Figure S6G). In addition, the protein levels of cleaved‐caspase9, cleaved‐caspase3, and Bax were remarkably reduced, and the protein levels of Bcl2 and XIAP were obviously enhanced in ATPIF1 knockdown mice compared with the WT group under I/R (Figure 6H). And the leakage level of cytochrome was enhanced (Figure 6I). The above data indicated that knockdown of ATPIF1 alleviated mitochondrial apoptosis and cardiac I/R injury.

### 2.8 METTL1 Regulates Mitochondrial Dysfunction by ATPIF1

**FIGURE 7:**
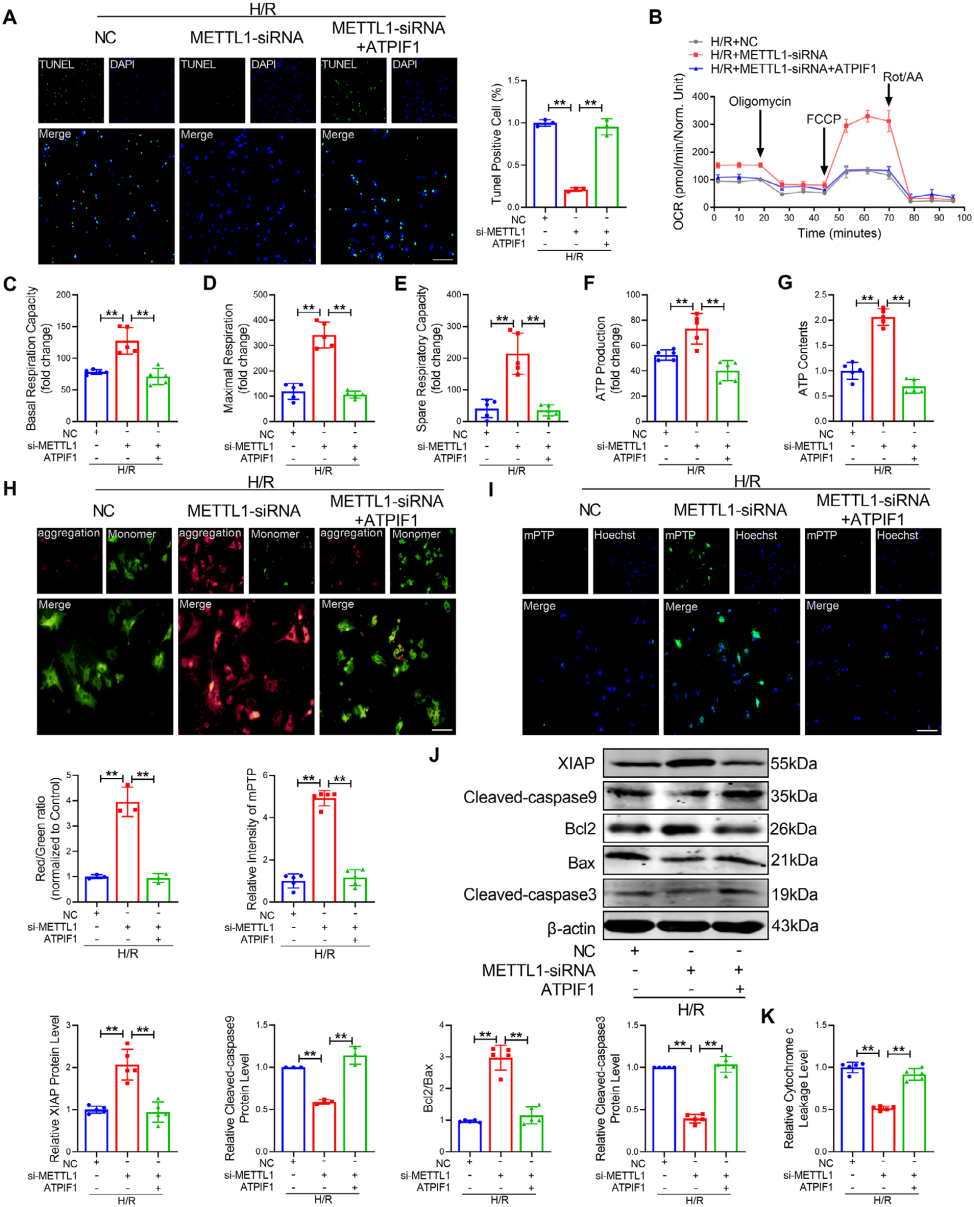
METTL1 regulated mitochondrial apoptosis and dysfunction via ATPIF1. (A) Apoptosis of CMs determined by TUNEL staining. Scale bar: 50 µm. *n* = 5. (B) Quantitative statistical analysis of OCR in CMs with co‐transfection of METTL1‐siRNA and ATPIF1‐pcDNA3.1 after H/R injury. *n* = 5. (C) Quantitative statistical analysis of basal respiration in CMs. *n* = 5. (D) Quantitative statistical analysis of maximal respiration in CMs. *n* = 5. (E) Quantitative statistical analysis of spare respiratory capacity in CMs. *n* = 5. (F) Quantitative statistical analysis of ATP production in CMs. *n* = 5. (G) Quantitative statistical analysis of ATP content in CMs*. n* = 5. (H) JC‐1 staining in CMs was used to detect mitochondrial membrane potential. Scale bar: 50 µm. *n* = 3. (I) MPTP staining in CMs was performed to detect mitochondrial permeability. Scale bar: 50 µm. *n* = 5. (J) The protein levels of Bcl2, Bax, XIAP, cleaved‐caspase9, and cleaved‐caspase3 by western blot. Bcl2, Bax, XIAP, and cleaved‐caspase3. *n* = 5. Cleaved‐caspase9. *n* = 3. (K) The leakage level of cytochrome c. *n* = 5. Values represent the mean ± SD. ^*^ *p* < 0.05, ^**^ *p* < 0.01.

Recognizing that METTL1 regulates the expression of ATPIF1 by mediating m^7^G tRNA modification, and ATPIF1 plays a crucial role in cardiac injury. We believed that ATPIF1 might be a downstream regulator of METTL1. Therefore, we investigated whether ATPIF1 could eliminate the effect of METTL1 during cardiac injury by performing rescue experiments. We first determined the overexpression efficiency of ATPIF1‐pcDNA3.1; the expression level of ATPIF1 was markedly raised after transfection with ATPIF1‐pcDNA3.1 (Figure S7A). We then co‐transfected ATPIF1‐pcDNA3.1 and METTL1‐siRNA into the CMs. TUNEL assays demonstrate that ATPIF1 abrogated the protective effect of the knockout of METTL1 on cardiomyocytes under H/R stimulation (Figure 7A). Furthermore, transfection of METTL1‐siRNA alone maintains myocardial cell energy metabolism under H/R insult, but the beneficial effect was countered by co‐transfection with the ATPIF1 plasmid, which included OCR, basal respiration, ATP production, spare respiratory capacity, maximal respiration, and ATP content (Figure 7B–G). Similarly, overexpression of ATPIF1 strikingly damaged mitochondrial membrane potential, opened mPTP, and enhanced mitochondrial superoxide under transfection of METTL1‐siRNA (Figure 7H,I; Figure S7B). Meanwhile, ATPIF1 promotes the expression of mitochondrial fission proteins Drp1 and Fis1 while inhibiting the expression of mitochondrial fusion proteins OPA1 and Mfn2 (Figure S7C). Importantly, ATPIF1 abolished the inhibitory effect of METTL1‐siRNA on mitochondrial apoptosis in cardiomyocytes, leading to reduced XIAP expression, decreased Bcl2/Bax ratio, elevated levels of cleaved‐caspase9 and cleaved‐caspase3, and exacerbated cytochrome c leakage (Figure 7J,K). These data suggest that METTL1 regulates mitochondrial dysfunction by ATPIF1.

### 2.9 ATPIF1 Mediates the Effects of METTL1 on Cardiac I/R Injury

**FIGURE 8:**
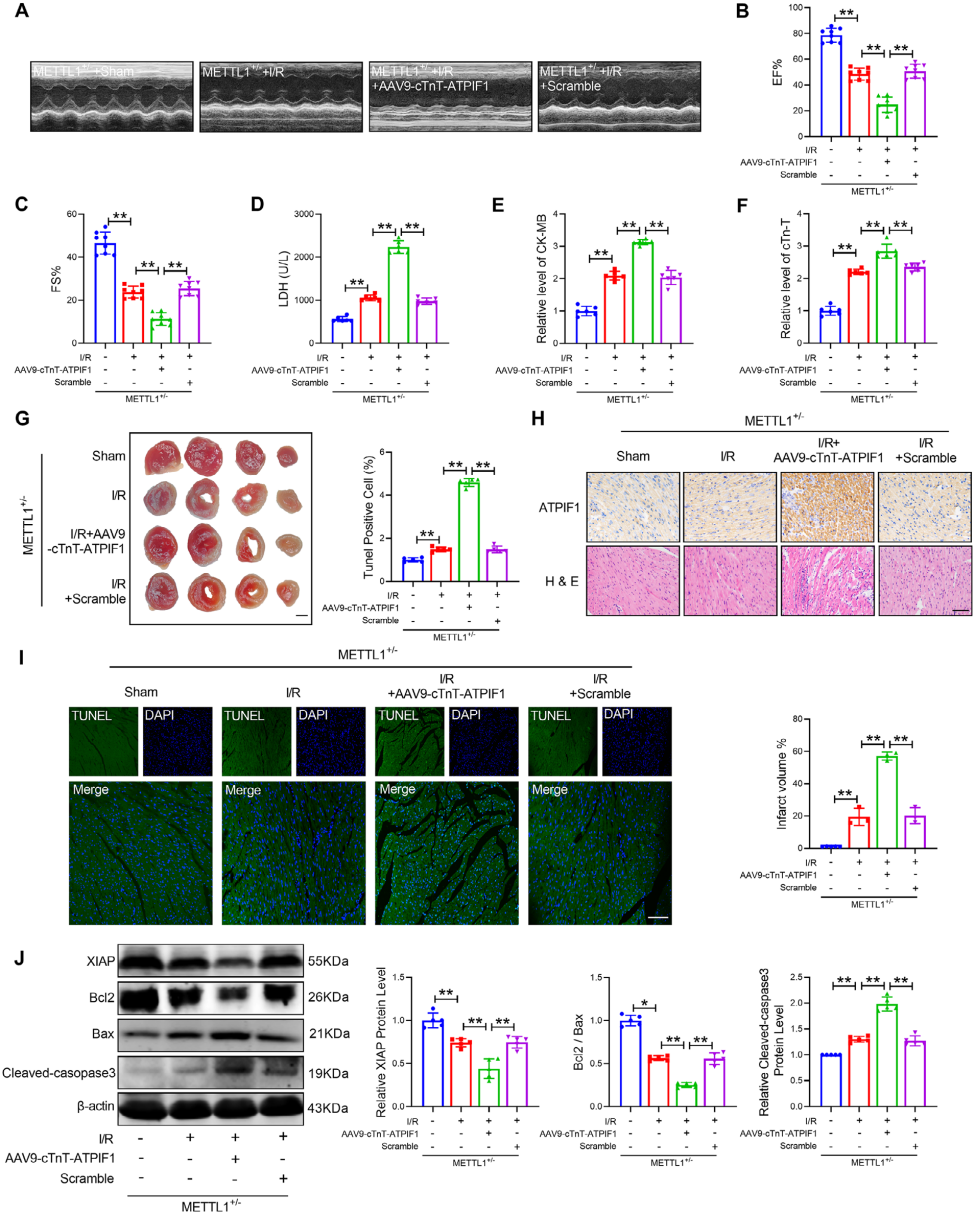
ATPIF1 abrogates the effects of METTL1 knockdown on cardiac ischemia–reperfusion injury. (A‐C) Cardiac function of METTL1^±^ mice and METTL1^+/−^ mice with AAV9‐cTnT‐ATPIF1 after I/R injury. *n* = 8. (D) Level of LDH in serum. *n* = 6. (E) The level of CK‐MB in serum. *n* = 6. (F) The level of cTn‐T in serum. *n* = 6. (G) Infarct size of I/R hearts by TTC staining. Scale bar: 2 mm. *n* = 3. (H) H&E and immunohistochemical detection of ATPIF1 in the left ventricles. Scale bars: 50 µm. *n* = 5. (I) TUNEL staining was used to identify cardiomyocyte apoptosis in the border zone. Scale bar: 20 µm. *n* = 5. (J) The protein levels of XIAP, Bcl2, Bax, and cleaved‐caspase3. *n* = 5. Values represent the mean ± SD. ^*^ *p* < 0.05, ^**^ *p* < 0.01.

To obtain more evidence to confirm that ATPIF1 mediates the effects of METTL1 on cardiac injury. We overexpressed ATPIF1 in METTL1^+/−^ mice by tail vein injection of the AAV9‐cTnT‐ATPIF1 and performed the I/R surgery on mice. Overexpression of ATPIF1 remarkably inhibited the protective effect of METTL1 knockout on I/R injury hearts, manifested as a decrease in cardiac function, an increase in infarct size, and an upregulation in the level of LDH, CK‐MB, and cTn‐T (Figure 8A–G). Meanwhile, overexpression of ATPIF1 also led to myocardial dysregulation and inflammatory infiltration (Figure 8H). We subsequently investigated whether ATPIF1 determines the extent of mitochondrial apoptosis following METTL1 knockout. Notably, increased ATPIF1 expression resulted in a significant elevation in TUNEL‐positive cells, levels of cleaved‐caspase3 and cleaved‐caspase9, and cytochrome c leakage, accompanied by a reduced Bcl‐2/Bax ratio and decreased XIAP expression (Figure 8I,J; Figure S8A,B). Consistently, ATPIF1 promotes the expression of mitochondrial fission proteins Drp1 and Fis1 while inhibiting the expression of fusion proteins OPA1 and Mfn2 (Figure S8B). These findings demonstrated that overexpression of ATPIF1 inhibits the improvement of cardiac I/R injury in METTL1^+/−^ mice.

## 3 Discussion

In the present study, we observed that (1) m^7^G tRNA modification and METTL1 were upregulated in H/R injury primary cardiomyocytes, I/R injury hearts, and the plasma of AMI patients; (2) inhibition of METTL1 improves mouse cardiac I/R injury; (3) mechanistically, knocking out METTL1 suppressed m^7^G tRNA methylation and the expression of the majority of m^7^G‐modified tRNAs, thereby downregulating the translation efficiency of ATPIF1 mRNA to restore the level of mitochondrial oxidative phosphorylation and ROS, ultimately inhibiting mitochondrial apoptosis and alleviating cardiac I/R injury; (4) both gain‐ and loss‐of‐function studies demonstrated that ATPIF1 knockdown alleviated I/R injury but ATPIF1 overexpression induced myocardial hypertrophy. These findings enhance our understanding of cardiac I/R injury with METTL1‐mediated m^7^G tRNA modifications as a novel molecular mechanism. They also propose METTL1 and ATPIF1 as promising therapeutic targets for treating I/R injury.

Epigenetic modification is closely related to the pathological progression of cardiac injury [31, 33, 34]. The tRNAs are the most highly modified RNA that recognize mRNA codons and transfer corresponding amino acids during protein synthesis. tRNAs are the dominant m^7^G‐modified RNA species [35, 36, 37]. The m^7^G tRNA modification is highly conserved among different species and has significant physiological functions [13]. A study has reported that m^7^G tRNA modification by METTL1 plays a crucial role in the process of self‐renewal and differentiation of mouse embryonic stem cells [16]. Furthermore, m^7^G tRNA modification influences oncogenic mRNA, potentially promoting the progression of leukemia, hepatocellular carcinoma, lung cancer, and intrahepatic cholangiocarcinoma [17, 38, 39]. In cardiovascular diseases, fibroblast‐specific deletion of METTL1 notably alleviated the myocardial infarction‐induced cardiac fibrosis by enhancing α‐SMA, col1, and col3 mRNA translation efficiency [24]. The interaction between TMEM11 and METTL1 was found to enhance the m^7^G methylation of Atf5 mRNA, thus regulating cardiomyocyte proliferation and contributing to cardiac repair [23]. There is still more to understand about how m^7^G tRNA modification affects cardiac I/R injury. In the study, we found m^7^G tRNA modification, and METTL1 were upregulated with cardiac I/R injury. Knockout of METTL1 alleviated cardiac I/R injury through restoring mitochondrial function and inhibiting cardiomyocyte mitochondrial apoptosis.

Moreover, we found that METTL1 can influence the expression of m^7^G‐modified tRNA. The m^7^G‐modified tRNAs were dramatically decreased in METTL1 knockdown cardiomyocytes. It has been reported previously that dysregulation of tRNA modifications and expression is linked to diseases [11, 12, 40, 41]. The involvement of oncogenes and tumor suppressor signaling pathways in the translational regulation of malignancies is mediated through the modulation of tRNA production [42, 43, 44, 45]. There is also evidence that alterations in individual tRNA also induce diseases. For example, the promotion of a pro‐metastatic state in breast cancer was facilitated by the overexpression of tRNA Glu‐UCC or Arg‐CCG [46]. For m^7^G‐modified tRNAs, it has been found that their dysregulation will affect the pause and occupation of ribosomes on the decoding codon of m^7^G‐modified tRNA [14, 39]. Therefore, we believe that depletion of METTL1 causes a reduction in m^7^G tRNA modification and the levels of m^7^G‐modified tRNAs, thereby increasing the pause and occupation of ribosomes on the decoding codon of m^7^G‐modified tRNA, leading to decreased global translation and reduced translation efficiency of target mRNAs through an m^7^G‐related codon‐dependent manner during I/R injury.

The results of the gene set enrichment assay indicated that a high enrichment of mRNAs with decreased translation efficiency in pathways related to mitochondrial and ATP synthesis. Mitochondria have been confirmed as crucial contributors to myocardial I/R injury [47], which induces cardiomyocyte death through the initiation of apoptosis in the reperfusion myocardium [8, 48]. Mitochondria supply over 90% of the energy needed for cardiomyocytes. Furthermore, various pathological conditions, such as immune responses, endoplasmic reticulum stress, oxidative stress, and calcium overload, are initiated, interconnected, or exacerbated by mitochondrial dysfunction. In instances of myocardial injury, mitochondria can safeguard their structure and function by activating mitochondrial quality control, ensuring an uninterrupted energy supply for damaged myocardial cells [49]. Our investigation revealed that silencing METTL1 diminishes the translation level of ATPIF1, thereby restoring mitochondrial oxidative phosphorylation function and ATP content, and mitigating the increase in mitochondrial ROS resulting from mitochondrial damage. It hindered the abnormal opening of the mPTP induced by ROS overload, consequently reducing calcium ion influx and reinstating mitochondrial membrane potential. Finally, mitochondrial dysfunction and mitochondrial apoptosis were inhibited.

Current research has highlighted the significance of ATPIF1 in cardiovascular disease [28, 50]. Nevertheless, the role of ATPIF1 in cardiovascular disease is currently inconsistent. It has been observed that the AMPK signaling pathway is activated by the upregulation of ATPIF1 in I/R heart, exerting cardioprotective effects [51]. Conversely, there was also a literature report that ATPIF1 was thought not to interact with complex V in normal circumstances, meaning that mitochondrial ability to synthesize ATP was unaffected. Importantly, the study conducted by Tian's research team revealed that mice with ATPIF1 deletion exhibited protection against cardiac dysfunction and pathological development caused by transverse aortic constriction or isoproterenol infusion [29]. The uncertain role of ATPIF1 is not conducive to the study of this protein. In this study, we separately established ATPIF1 overexpression and knockdown mouse models. The data indicate that knockdown of ATPIF1 alleviates cardiac I/R injury, while overexpression of ATPIF1 induces myocardial hypertrophy. Additionally, our observations in cardiomyocytes with H/R reveal that knocking down ATPIF1 contributes to inhibiting mitochondrial ROS and maintaining mitochondrial homeostasis. This study contributes new evidence to elucidate the role of ATP1F in cardiovascular disease.

Despite the meaningful findings of this study, several limitations remain. First, although we detected elevated serum levels of METTL1 and ATPIF1 in AMI patients, the collection of clinical specimens was inherently challenging due to strict inclusion criteria and limited access to eligible participants. This constraint has limited the sample size to some extent. Therefore, future studies should aim to expand the clinical cohort when possible to strengthen the robustness of our conclusions. Second, we found that METTL1 not only regulates ATPIF1 expression but may also influence other mitochondrial and oxidative phosphorylation‐related genes, such as SLC25A39 and ATP5O. Both protein and mRNA levels of these genes showed significant alterations upon METTL1 knockout, suggesting that the underlying mechanisms may extend beyond m7G modification. In future work, we will further explore these potential mechanisms and propose plausible explanations.

In summary, we uncovered for the first time that METTL1 regulates myocardial I/R injury through mediating m^7^G tRNA modification. Silencing METTL1 suppressed m^7^G tRNA methylation, which in turn decreased the translation efficiency of ATPIF1 mRNA. This ultimately led to regulating mitochondrial energy metabolism and reducing mitochondrial ROS and apoptosis levels, thereby alleviating cardiac I/R injury. These findings provide novel insights into the role of m^7^G tRNA modification in cardiac I/R injury. Consequently, METTL1 and ATPIF1 may serve as potential therapeutic targets for treating cardiac I/R injury.

## 4 Materials and Methods

### 4.1 Human Plasma Samples

11 AMI patients and 11 non‐AMI control participants contributed their plasma samples taken from the Sun Yat‐sen Memorial Hospital (Guangzhou, China), between June 2023 and September 2023. AMI was identified by combining a number of criteria, such as ischemic symptoms, the presence of an abnormal Q wave, elevated cardiac troponin I and CKMB levels, and ST‐segment depression or elevation as defined by the European Society of Cardiology/American College of Cardiology. The use of human samples was approved by the institutional review board of Sun Yat‐sen University (protocol SYSKY‐2024‐1146‐01) and conformed to the Declaration of Helsinki. Written informed consent was obtained from each of the participants.

### 4.2 Animals and Treatment

Male C57BL/6 mice, aged eight weeks, were purchased from Zhuhai BesTest Bio‐Tech Co, Ltd, China. They were housed in a constant environmental condition with 12 h of light and dark cycles and unlimited access to food and water. METTL1^+/−^ mice are generated by using CRISPR/Cas9‐mediated genome editing (Cyagen, Hangzhou, China). Age‐paired wild‐type and METTL1^+/−^ mice are used in subsequent experiments. AAV9‐cTnT‐shATPIF1, AAV9‐cTnT‐ATPIF1, and their negative control were obtained from GENECHEM (Shanghai, China). WT mice and METTL1^+/−^ mice were injected with 0.1 mL AAV9 through the tail vein at a dose of 5 × 10^12^ vg/kg. All animal care and protocol of experiments were approved by the Guangdong Pharmaceutical University Committee on Animal Care (protocol gdpulacspf2022106). To an I/R injury, 8‐week‐old mice were treated to ligation/perfusion of the left anterior descending coronary artery (LAD). The heart was exposed after Tribromoethanol anesthesia, and the LAD was ligated with a 7‐0 silk knot. After 45 min of ischemia, LAD was allowed to restore blood flow for 24 h and release the live knot. After reperfusion, blood was collected, and the heart was quickly removed.

### 4.3 Mouse Neonatal Cardiomyocyte Isolation and Culture

CMs were extracted from 1–3‐day‐old mice. Isolated hearts were washed and digested in pancreatin (Biosharp, BL501A, China). After centrifugation for 5 min, cells were resuspended in DMEM (Corning, 10013CV, USA). Following the manufacturer's instructions, CMs were transfected with plasmids and small interfering RNA (siRNA) using Lipofectamine 2000 (Invitrogen, 11668019, USA). After transfection for 48 h, CMs were treated with 200 µM H_2_O_2_ (Sigma‐Aldrich, 323381, USA) for 1 h and reoxygenation for 24 h to establish a hypoxia/reoxygenation cell model. The ATPIF1 plasmids and their negative control vectors were provided by BT Lab (Wuhan, China). METTL1‐siRNA and ATPIF1‐siRNA were provided by Ribo Bio (Guangzhou). The sequence of METTL1 siRNA was 5’‐GGATGTGCACTCATTTCGA‐3’, and ATPIF1 siRNA was 5’‐GAAGATCCAAC AACTAAAG‐3’.

### 4.4 Northern Blot and Northwestern Blot

For northern blot of U6 snoRNA, 4 µg total RNA samples were separated by 15 % TBE‐urea gel according to molecular weight. RNA was transferred to a positively charged nylon membrane and cross‐linked with an ultraviolet cross‐linking instrument for 3 min. Digoxin‐labeled U6 snRNA was used for imprinting. Northwestern blotting, using anti‐m^7^G antibody for RNA‐containing membrane imprinting. Digoxin and anti‐m^7^G antibody signals were detected. The sequence of U6 snRNA was 5′‐TGGAACGCTTCACGAATTTG‐3′.

### 4.5 Analysis of Oxygen Consumption Rate

According to the manufacturer's recommended protocol, OCR was analyzed by the Seahorse XFe24 analyzer (Agilent Technologies, California, USA). In short, the cells were added to the hippocampal cell culture microplate before measurement. After transfection and H/R treatment, DMEM was removed and replaced with Seahorse XF DMEM culture. Drugs: oligomycin (1.5 µM), rotenone/antimycin A (0.5 µM), and FCCP (4 µM) were added in turn to monitor OCR in real time. All reagents are from Agilent Technologies. Finally, the cells in the culture plate were digested with EDTA, and the cells were counted. The results were analyzed by Seahorse Wave Desktop 2.6.3.5 (Agilent).

### 4.6 Puromycin Intake Assay

Puromycin with a final concentration of 1 µM was added to the cells and incubated in the 37°C cell incubator for 30 min. The cells were lysed with RIPA lysate to extract protein, followed by a western blot, and the level of puromycin intake was detected by anti‐puromycin antibody.

### 4.7 Western Blot Analyses

Protein expression was evaluated by the western blot analysis. Total protein was extracted using RIPA lysis buffer. BCA protein assay kit (Beyotime, P0011‐1, China) was used to determine the protein concentration. After being electrophoresed through 13% SDS‐PAGE, proteins were transferred to NC membranes (PALL, 66485, USA). After blocking with 5% skim milk at room temperature, membranes were incubated with the designated primary antibodies against various antigens, such as METTL1, WDR4, ATPIF1, Fis1, Drp1, Opa1, Mfn2, Bcl2, Bax, XIAP, cleaved‐caspase9, cleaved‐caspase3, and β‐actin, for overnight at 4°C. Next, membranes were incubated at room temperature for one hour with a secondary antibody (1:8000). Blots were detected and analyzed using the Odyssey Imaging System (Gene Company Ltd, Hong Kong, China). The antibodies used in this study are presented in Table S1.

### 4.8 Reverse Transcription and Quantitative Real‐Time PCR

According to the manufacturer's protocol, total RNA was extracted with Trizol. Following that, they were reverse‐transcribed into cDNA. qRT‐PCR was used to quantify the expression of the target gene mRNA using SYBR Green Master Mix. And the β‐actin was used as an endogenous control. The sequences of primer pairs used in this study are presented in Table S2.

Echocardiography, infarct size detection, hematoxylin–eosin and immunohistochemical staining of the heart tissues, ATP detection, flow cytometric analysis of apoptosis, MeRIP‐tRNA sequencing, tRNA sequencing, ribosome profiling sequencing, creatine kinase MB isoenzyme, cardiac troponin T, quantification of Cytochrome c release by ELISA, assessment of the mPTP opening in CMs, JC‐1 staining, TUNEL staining, Mitosox staining, wheat germ agglutinin, and lactate dehydrogenase methods are in the Supporting Information.

### 4.9 Statistical Analyses

The data in this study are presented as the mean ± SD of multiple independent replicates. Statistical comparisons were performed using Student's *t*‐test between two groups or one‐way ANOVA for multiple comparisons. *p* < 0.05 indicated a significant difference, and data were analyzed with the GraphPad Prism 8.

## Author Contributions

Conceptualization: J.G., H.L., Y.Z., M.L., and R.W. Designing the experiments: Y.Z., R.W., M.L., Q.L., and H.D. Performing the experiments and data analysis: M.L., R.W., X.T., Z.C., L.W., and X.S. Providing human plasma samples: Y.J., H.Z., X.L., and Y.W. Writing – original draft: Y.Z., R.W., and M.L. Writing – review & editing: M.L., X.T., and X.S. All of the authors have read and approved the final manuscript.

## Funding

This study was supported by Ministry of Science and Technology of the People's Republic of China [2023YFC3606200], National Natural Science Foundation of China (T2341005, 82570355, 82304482, 82204390), Department of Education of Guangdong Province [2023KCXTD019]; the Open Project Program of State Key Laboratory of Frigid Zone Cardiovascular Diseases (SKLFZCD), Harbin Medical University (no. HDHY2024016); the team project of “Subject Enrichment, Innovation and Quality Improvement” in Guangdong Pharmaceutical University (2024QZ08).

## Ethics Statement

The use of human samples was approved by the institutional review board of Sun Yat‐sen University (protocol SYSKY‐2024‐1146‐01) and conformed to the Declaration of Helsinki. Written informed consent was obtained from each of the participants. All animal care and protocol of experiments were approved by the Guangdong Pharmaceutical University Committee on Animal Care (protocol gdpulacspf2022106).

## Conflicts of Interest

The authors declare no conflicts of interest.

## Consent for Publication

The authors consent to publishing this work.

## Supporting information




**Figure S1**: Effects of myocardial I/R injury on cardiac function in mice. (A–C) Cardiac function evaluated by echocardiography from the parasternal short‐axis view in WT mice and WT mice after I/R injury. *n* = 8. (D) Hematoxylin‐eosin (H&E) and immunohistochemical staining of the left ventricles of mice. Scale bars: 50 µm. *n* = 5. Values represent the mean ± SD. ^**^ *p* < 0.01.
**Figure S2**: Silencing the expression of METTL1 inhibits cardiomyocyte apoptosis. (A) The mRNA level of METTL1 in wild type (WT) and METTL1^+/−^ mice upon I/R injury by qRT‐PCR. *n* = 6. (B) The protein level of METTL1 in wild type (WT) and METTL1^+/−^ mice upon I/R injury by western blot. *n* = 6. (C) The mRNA level of METTL1 in CM treated with H/R after transfection with METTL1‐siRNA. *n* = 6. (D) The protein level of METTL1 in CM treated with H/R after transfection with METTL1‐siRNA. *n* = 7. (E) Apoptosis of cardiomyocyte determined by TUNEL staining. Scale bar: 50 µm. *n* = 3. (F) The protein levels of Bax, Bcl2, XIAP, and cleaved‐caspase3. *n* = 4. XIAP, *n* = 5. Bax, Bcl2, and cleaved‐caspase3. *n* = 3. Values represent the mean ± SD. ^*^ *p* < 0.05, ^**^ *p* < 0.01.
**Figure S3**: Silencing the expression of METTL1 inhibits mitochondrial dysfunction. (A) Effects of METTL1 knockdown on ATP content in CM. *n* = 5 (B) Mitosox staining in CMs was used to detect mitochondrial ROS. Scale bar: 50 µm. *n* = 3. (C) The protein levels of OPA1, Mfn2, Drp1, and Fis1 in wild‐type (WT) and METTL1± mice upon I/R injury were determined by western blot. *n* = 5. (D) The protein levels of OPA1, Mfn2, Drp1, and Fis1 in CM treated with H/R after transfection with METTL1‐siRNA. *n* = 5. (E) The protein levels of cleaved‐caspase9. *n* = 3. (F) The leakage level of cytochrome c. *n* = 5. Values represent the mean ± SD. ^*^ *p* < 0.05, ^**^ *p* < 0.01.
**Figure S4**: METTL1 regulates m^7^G tRNA modification, tRNA expression, and mRNA translation. (A) Northwestern blot of m^7^G. U6 snRNA was used as a loading control. *n* = 4. (B) Expression profile of m^7^G modification in the METTL1 knockdown and WT mice with I/R injury by MeRIP‐tRNA‐seq. (C) Puromycin intake assay of CMs with METTL1‐siRNA or METTL1‐pcDNA3.1. *n* = 5. Values represent the mean ± SD. ^*^ *p* < 0.05, ^**^ *p* < 0.01.
**Figure S5**: ATPIF1 may be a key target in METTL1‐mediated mitochondrial damage and myocardial injury. (A) The protein levels of ATP5G3 in wild type (WT) and METTL1^+/−^ mice upon I/R injury by Western blot. *n* = 3. (B) The mRNA level of ATPIF1, ATP5G3, and SLC25A3 in wild type (WT) and METTL1^+/−^ mice upon I/R injury by qRT‐PCR. *n* = 6. (C) Immunohistochemical staining of mice left ventricles. Scale bars: 50 µm. *n* = 5. (D) The protein levels of ATPIF1 in heart tissues subjected to I/R. *n* = 6. (E) The protein levels of ATPIF1 in CM subjected to H/R. *n* = 6. Values represent the mean ± SD. ^*^ *p* < 0.05, ^**^ *p* < 0.01.
**Figure S6**: Knockdown of ATPIF1 alleviates mitochondrial dysfunction. (A) qRT‐PCR analyzed the mRNA expression of ATPIF1 in heart with transfection of AAV9‐cTnT‐shATPIF1. *n* = 6. (B) The level of CK‐MB in serum. *n* = 6. (C) The level of cTn‐T in serum. *n* = 6. (D) qRT‐PCR analyzed the mRNA expression of ATPIF1 in CMs with transfection of ATPIF1‐siRNA. *n* = 6. (E) JC‐1 staining in CMs was used to detect mitochondrial membrane potential. Scale bar: 50 µm. *n* = 3. (F) MPTP staining in CMs was performed to detect mitochondrial permeability. Scale bar: 50 µm. *n* = 5. (G) Mitosox staining in CMs was used to detect mitochondrial ROS. Scale bar: 50 µm. *n* = 3. Values represent the mean ± SD. ^**^ *p* < 0.01.
**Figure S7**: ATPIF1 blocks the regulation of METTL1 on mitochondrial dysfunction. (A) qRT‐PCR analyzed the mRNA expression of ATPIF1 in CMs with transfection of the ATPIF1 plasmid. *n* = 6. (B) Mitosox staining in CMs was used to detect mitochondrial ROS. Scale bar: 50 µm. *n* = 3. (C) The protein levels of OPA1, Mfn2, Drp1, Fis1, and ATPIF1 in CMs. *n* = 5. Values represent the mean ± SD. ^*^ *p* < 0.05, ^**^ *p* < 0.01.
**Figure S8**: ATPIF1 reversed the therapeutic effect of knockdown METTL1. (A) The protein levels of cleaved‐caspase9 in METTL1^+/−^ mice and METTL1^+/−^ mice with AAV9‐cTnT‐ATPIF1 after I/R injury by western blot. *n* = 5. (B) The leakage level of cytochrome c. *n* = 5. (C) The protein levels of OPA1, Mfn2, Drp1, Fis1, and ATPIF1. *n* = 3. Values represent the mean ± SD. ^*^ *p* < 0.05, ^**^ *p* < 0.01.
**Table S1**: Antibodies used in this study.
**Table S2**: Primers used for the qRT‐PCR assay.

## Data Availability

The datasets used and/or analyzed during the current study are available from the corresponding author on reasonable request.
