# Supplementary material for: METTL1‐Mediated N7‐Methylguanosine tRNA Modification Alleviates Cardiac Ischemia/Reperfusion Injury by Modulating Mitochondrial Energy Metabolism
Source: MedComm (2020). 2026 Jan 7;7(1):e70572. doi: 10.1002/mco2.70572 (PMC12778400; doi:10.1002/mco2.70572)
Supplement: Supplementary file 1 — Figure S1: Effects of myocardial I/R injury on cardiac function in mice. (A–C) Cardiac function evaluated by echocardiography from the parasternal short‐axis view in WT mice and WT mice after I/R injury. n = 8. (D) Hematoxylin‐eosin (H&E) and immunohistochemical staining of the left ventricles of mice. Scale bars: 50 µm. n = 5. Values represent the mean ± SD. ** p < 0.01. Figure S2: Silencing the expression of METTL1 inhibits cardiomyocyte apoptosis. (A) The mRNA level of METTL1 in wild type (WT) and METTL1+/− mice upon I/R injury by qRT‐PCR. n = 6. (B) The protein level of METTL1 in wild type (WT) and METTL1+/− mice upon I/R injury by western blot. n = 6. (C) The mRNA level of METTL1 in CM treated with H/R after transfection with METTL1‐siRNA. n = 6. (D) The protein level of METTL1 in CM treated with H/R after transfection with METTL1‐siRNA. n = 7. (E) Apoptosis of cardiomyocyte determined by TUNEL staining. Scale bar: 50 µm. n = 3. (F) The protein levels of Bax, Bcl2, XIAP, and cleaved‐caspase3. n = 4. XIAP, n = 5. Bax, Bcl2, and cleaved‐caspase3. n = 3. Values represent the mean ± SD. * p < 0.05, ** p < 0.01. Figure S3: Silencing the expression of METTL1 inhibits mitochondrial dysfunction. (A) Effects of METTL1 knockdown on ATP content in CM. n = 5 (B) Mitosox staining in CMs was used to detect mitochondrial ROS. Scale bar: 50 µm. n = 3. (C) The protein levels of OPA1, Mfn2, Drp1, and Fis1 in wild‐type (WT) and METTL1± mice upon I/R injury were determined by western blot. n = 5. (D) The protein levels of OPA1, Mfn2, Drp1, and Fis1 in CM treated with H/R after transfection with METTL1‐siRNA. n = 5. (E) The protein levels of cleaved‐caspase9. n = 3. (F) The leakage level of cytochrome c. n = 5. Values represent the mean ± SD. * p < 0.05, ** p < 0.01. Figure S4: METTL1 regulates m7G tRNA modification, tRNA expression, and mRNA translation. (A) Northwestern blot of m7G. U6 snRNA was used as a loading control. n = 4. (B) Expression profile of m7G modification in the M [file MCO2-7-e70572-s001.docx]

**Supplemental**

**METTL1-mediated N^7^-methylguanosine tRNA modification** **alleviates cardiac ischemia/reperfusion injury by modulating mitochondrial energy metabolism**

Yue Zhang ^1#^, Mingyang Leng ^1#^, Ruonan Wang ^1#^, Xinyuan Tang ^1^, Zhenlu Cai ^1^, Liang Wang ^1^, Xiaoqi Shao ^1^, Hongtao Diao ^1^, Qinqiang long ^1^, Xu Li ^1^, Yingzi Wu ^1^, Yuan Jiang ^2^, Haifeng Zhang ^2^, Haihai Liang ^3, *^ and Jiao Guo ^1, *^

^1^ Key Laboratory of Glucolipid Metabolic Disorder, Ministry of Education of China; Guangdong Metabolic Diseases Research Center of Integrated Chinese and Western Medicine; Guangdong Key Laboratory of Metabolic Disease Prevention and Treatment of Traditional Chinese Medicine; Institute of Chinese Medicine, Guangdong Pharmaceutical University, Guangzhou, Guangdong Province, China; Key Unit of Modulating Liver to Treat Hyperlipemia SATCM, State Administration of Traditional Chinese Medicine.

^2^ Department of Cardiology, Sun Yat-sen Memorial Hospital, Sun Yat-sen University, Guangzhou, 510120, China.

^3^ State Key Laboratory of Frigid Zone Cardiovascular Diseases (SKLFZCD), Department of Pharmacology (State-Province Key Laboratories of Biomedicine-Pharmaceutics of China, Key Laboratory of Cardiovascular Research, Ministry of Education), College of Pharmacy, Harbin Medical University, Harbin 150081, China.

# These authors contributed equally to this work and are co-first authors.

* Corresponding authors.

Haihai Liang, Harbin Medical University, Harbin 150081, China; E-mail: [lianghaihai@ems.hrbmu.edu.cn](mailto:lianghaihai@ems.hrbmu.edu.cn)

Jiao Guo, Guangdong Pharmaceutical University, Guangzhou 510006, China; E-mail: [gyguoyz@163.com](file:///F:\METTL1数据\gyguoyz@163.com)

**Figure S1**


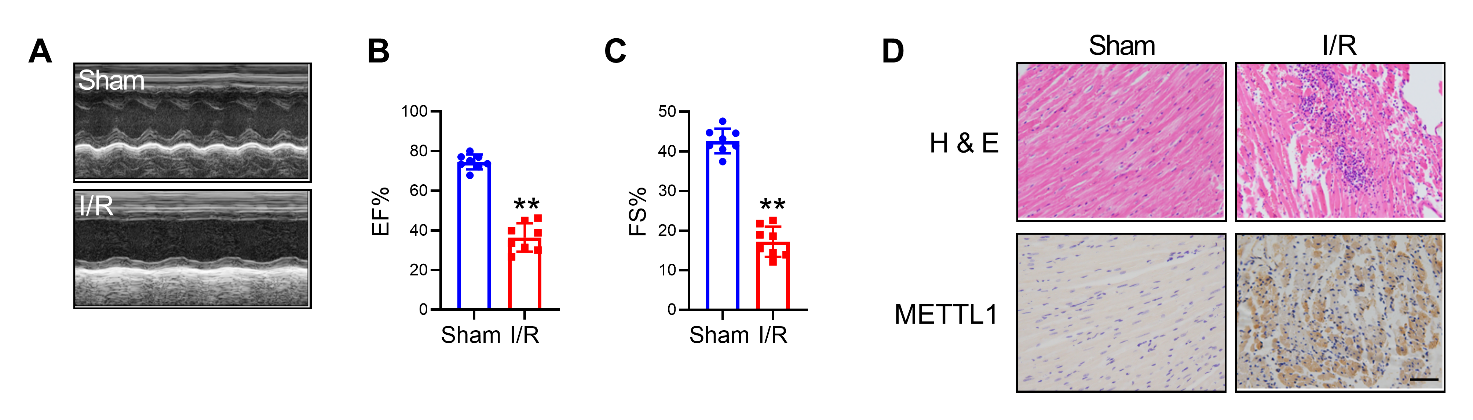


**Fig. S1** **Effects of myocardial I/R injury on cardiac function in mice. (A-C)** Cardiac function evaluated by echocardiography from the parasternal short-axis view in WT mice and WT mice after I/R injury. n=8. **(D)** Hematoxylin-eosin (H & E) and immunohistochemical staining of mice left ventricles. Scale bars: 50 μm. n=5. Values represent the mean ± SD. ^**^*P* < 0.01.

**Figure S2**


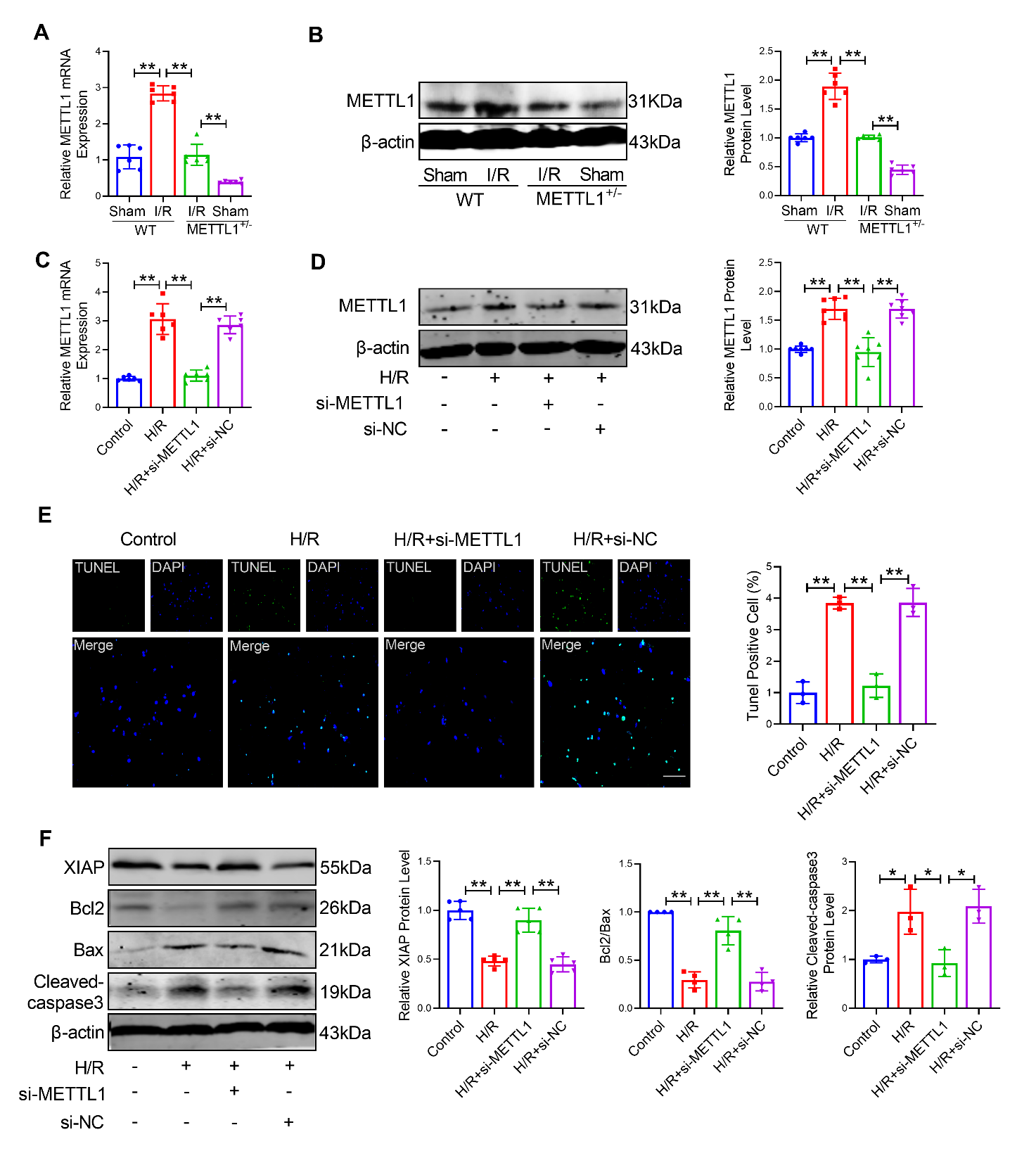


**Fig. S2 Silencing the expression of METTL1 inhibits cardiomyocyte apoptosis. (A)** The mRNA level of METTL1 in wild type (WT) and METTL1^+/-^ mice upon I/R injury by qRT-PCR. n=6. **(B)** The protein level of METTL1 in wild type (WT) and METTL1^+/-^ mice upon I/R injury by Western blot. n=6. **(C)** The mRNA level of METTL1 in CM treated with H/R after transfection with METTL1-siRNA. n=6. **(D)** The protein level of METTL1 in CM treated with H/R after transfection with METTL1-siRNA. n=7. **(E)** Apoptosis of cardiomyocyte determined by TUNEL staining. Scale bar: 50 μm. n=3. **(F)** The protein levels of Bax, Bcl2, XIAP and Cleaved-caspase3. n=4. XIAP, n=5. Bax, Bcl2 and Cleaved-caspase3. n=3. Values represent the mean ± SD. ^*^*P* < 0.05, ^**^*P* < 0.01.

**Figure S3**

**
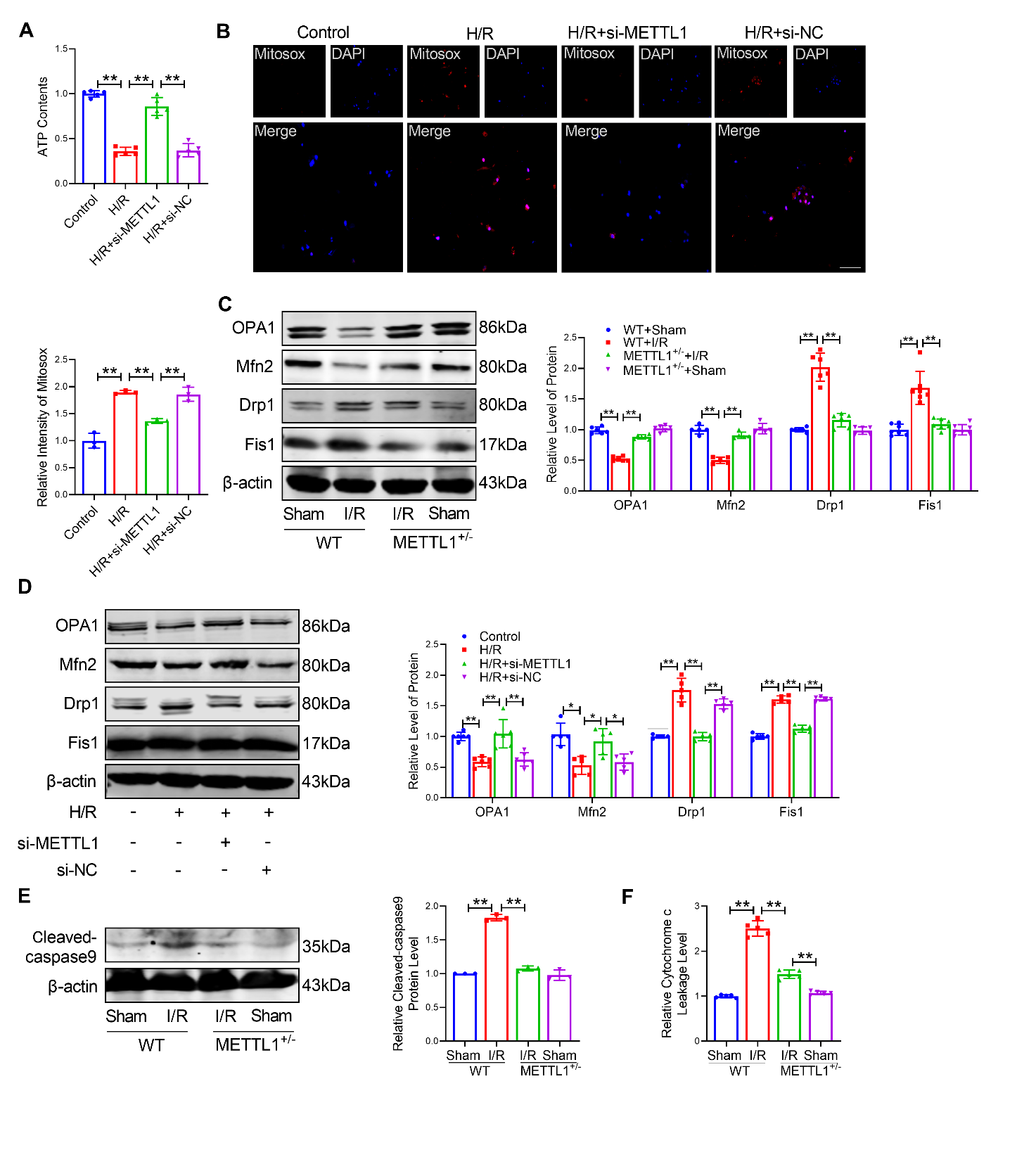
**

**Fig. S3 Silencing the expression of METTL1 inhibits mitochondrial dysfunction. (A)** Effects of METTL1 knockdown on ATP content in CM. n=5 **(B)** Mitosox staining in CMs was used to detect mitochondrial ROS. Scale bar: 50μm. n=3. **(C)** The protein levels of OPA1, Mfn2, Drp1, and Fis1 in wild type (WT) and METTL1+/- mice upon I/R injury by Western blot. n=5. **(D)** The protein levels of OPA1, Mfn2, Drp1, and Fis1 in CM treated with H/R after transfection with METTL1-siRNA. n=5. **(E)** The protein levels of Cleaved-caspase9. n=3. **(F)** The leakage level of cytochrome c. n=5. Values represent the mean ± SD. ^*^*P* < 0.05, ^**^*P* < 0.01.

**Figure S4**

**
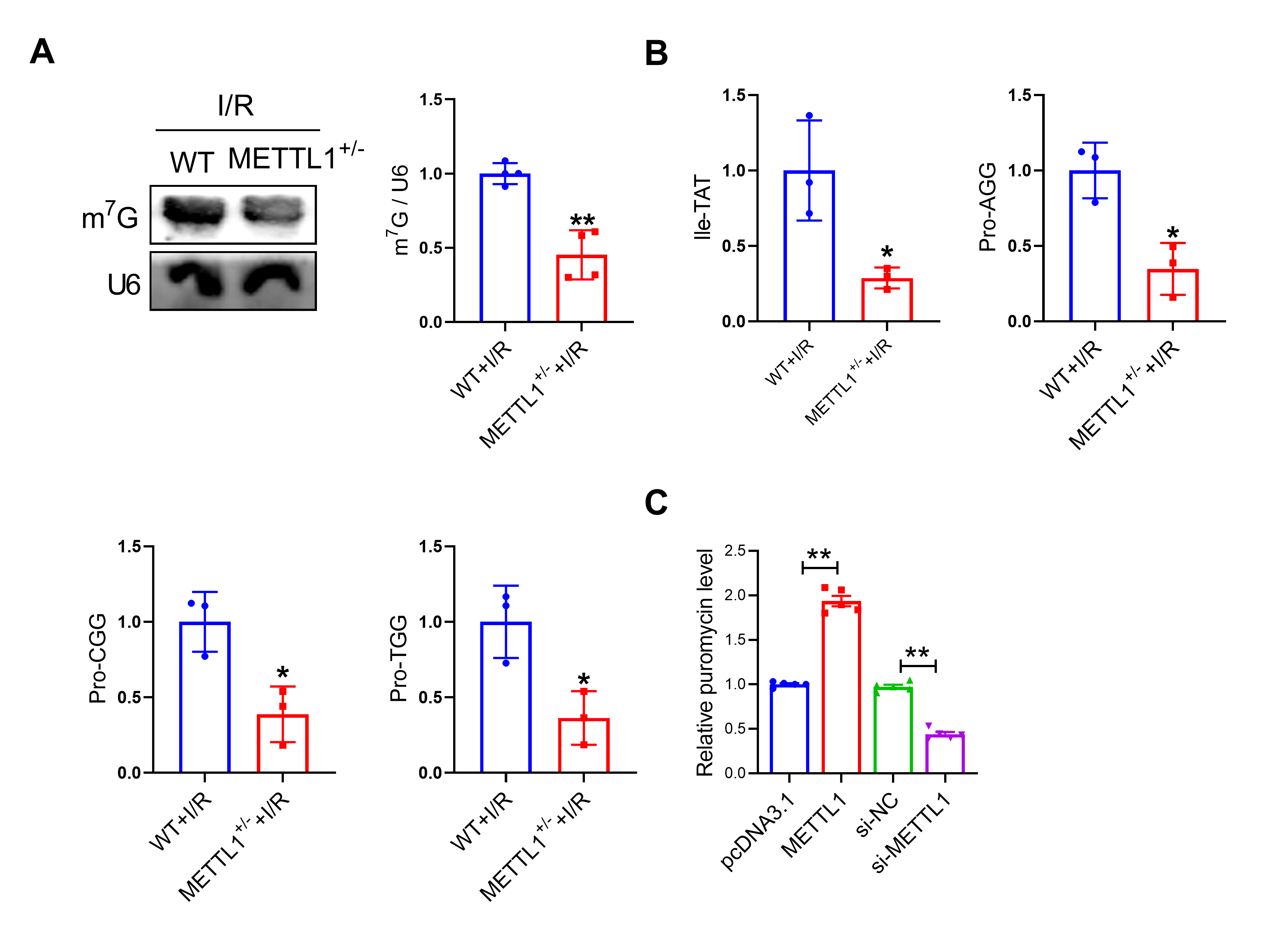
**

**Fig. S4 METTL1 regulates m^7^G tRNA modification, tRNA expression, and mRNA translation. (A)** Northwestern blot of m^7^G. U6 snRNA was used as a loading control. n=4. **(B)** Expression profile of m^7^G modification in the METTL1 knockdown and WT mice with I/R injury by MeRIP-tRNA-seq. **(C)** Puromycin intake assay of CMs with METTL1-siRNA or METTL1-pcDNA3.1. n=5. Values represent the mean ± SD. ^*^*P* < 0.05, ^**^*P* < 0.01.

**Figure S5**


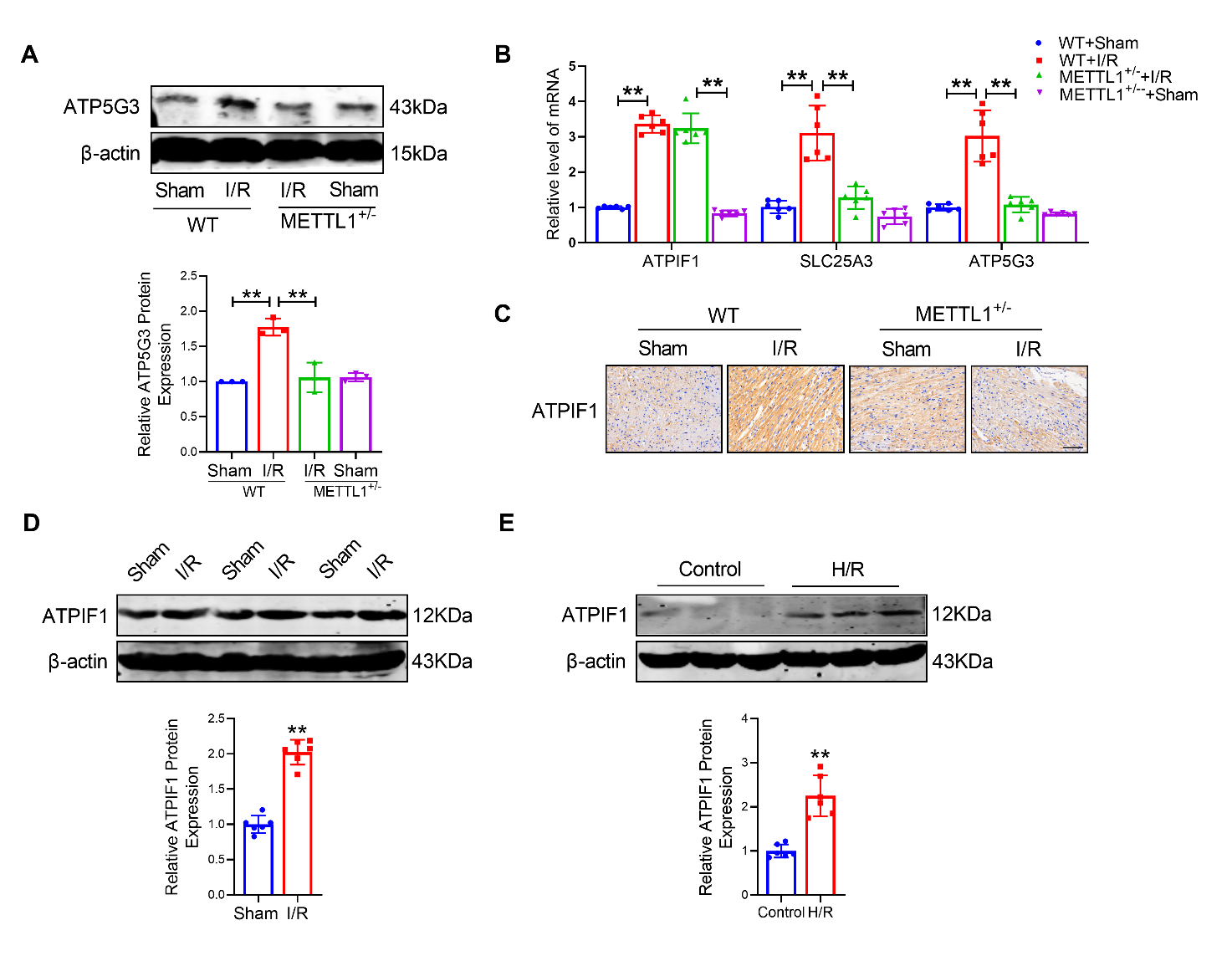


**Fig. S5 ATPIF1 may be a key target in METTL1-mediated mitochondrial damage and myocardial injury. (A**) The protein levels of ATP5G3 in wild type (WT) and METTL1^+/-^ mice upon I/R injury by Western blot. n=3. **(B)** The mRNA level of ATPIF1, ATP5G3, and SLC25A3 in wild type (WT) and METTL1^+/-^ mice upon I/R injury by qRT-PCR. n=6. **(C)** Immunohistochemical staining of mice left ventricles. Scale bars: 50 μm. n=5. **(D)** The protein levels of ATPIF1 in heart tissues subjected to I/R. n=6. **(E)** The protein levels of ATPIF1 in CM subjected to H/R. n=6. Values represent the mean ± SD. ^*^*P* < 0.05, ^**^*P* < 0.01.

**Figure S6**

**
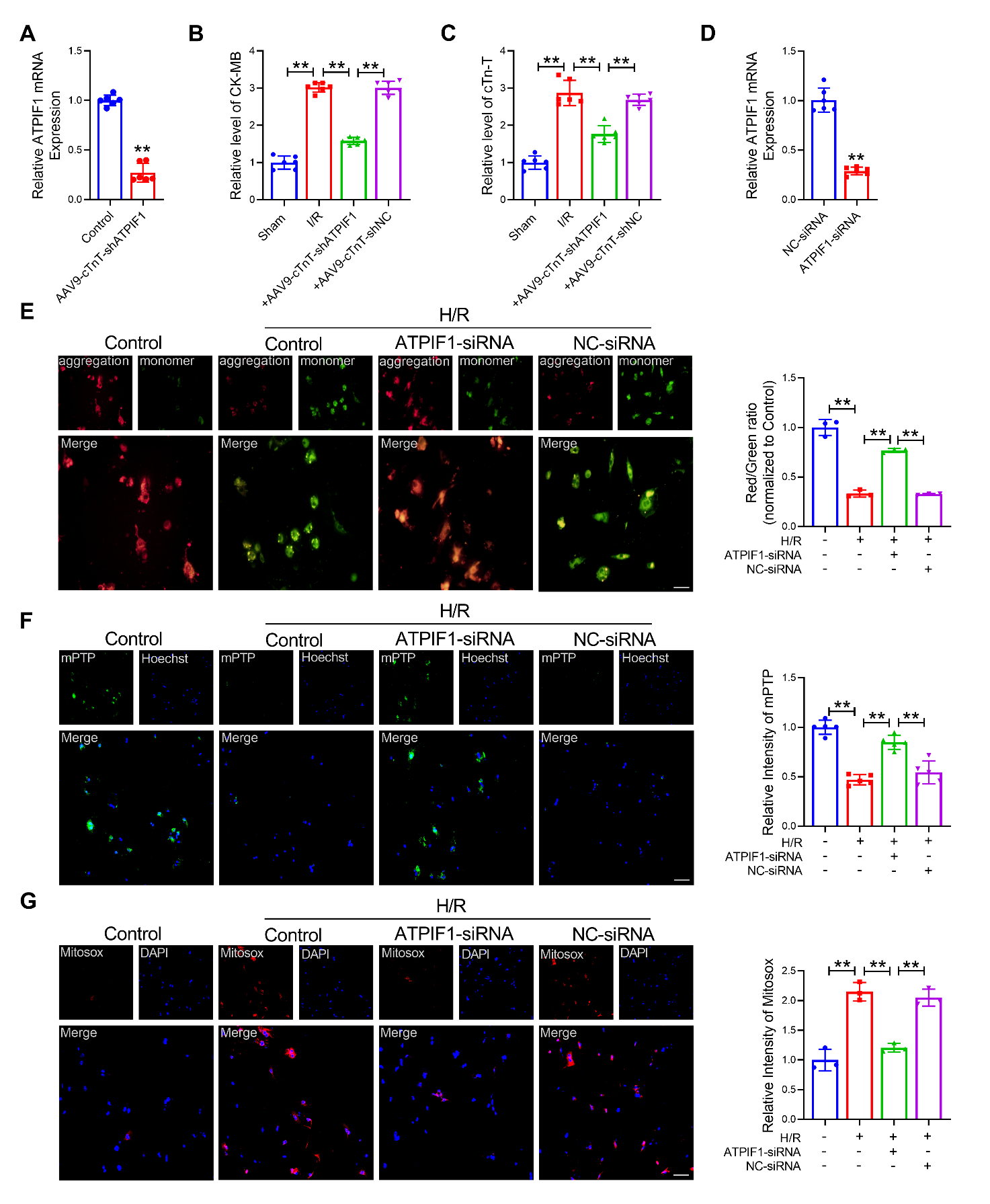
**

**Fig. S6 Knockdown of ATPIF1 alleviates mitochondrial dysfunction.** (**A**) qRT-PCR analyzed the mRNA expression of ATPIF1 in heart with transfection AAV9-cTnT-shATPIF1. n=6. (**B**) The level of CK-MB in serum. n=6. (**C**) The level of cTn-T in serum. n=6. (**D**) qRT-PCR analyzed the mRNA expression of ATPIF1 in CMs with transfection ATPIF1-siRNA. n=6. **(E)** JC-1 staining in CMs was used to detect mitochondrial membrane potential. Scale bar: 50 μm. n=3. **(F)** MPTP staining in CMs was performed to detect mitochondrial permeability. Scale bar: 50 µm. n=5. **(G)** Mitosox staining in CMs was used to detect mitochondrial ROS. Scale bar: 50μm. n=3. Values represent the mean ± SD. ^**^*P* < 0.01.

**Figure S7**

**
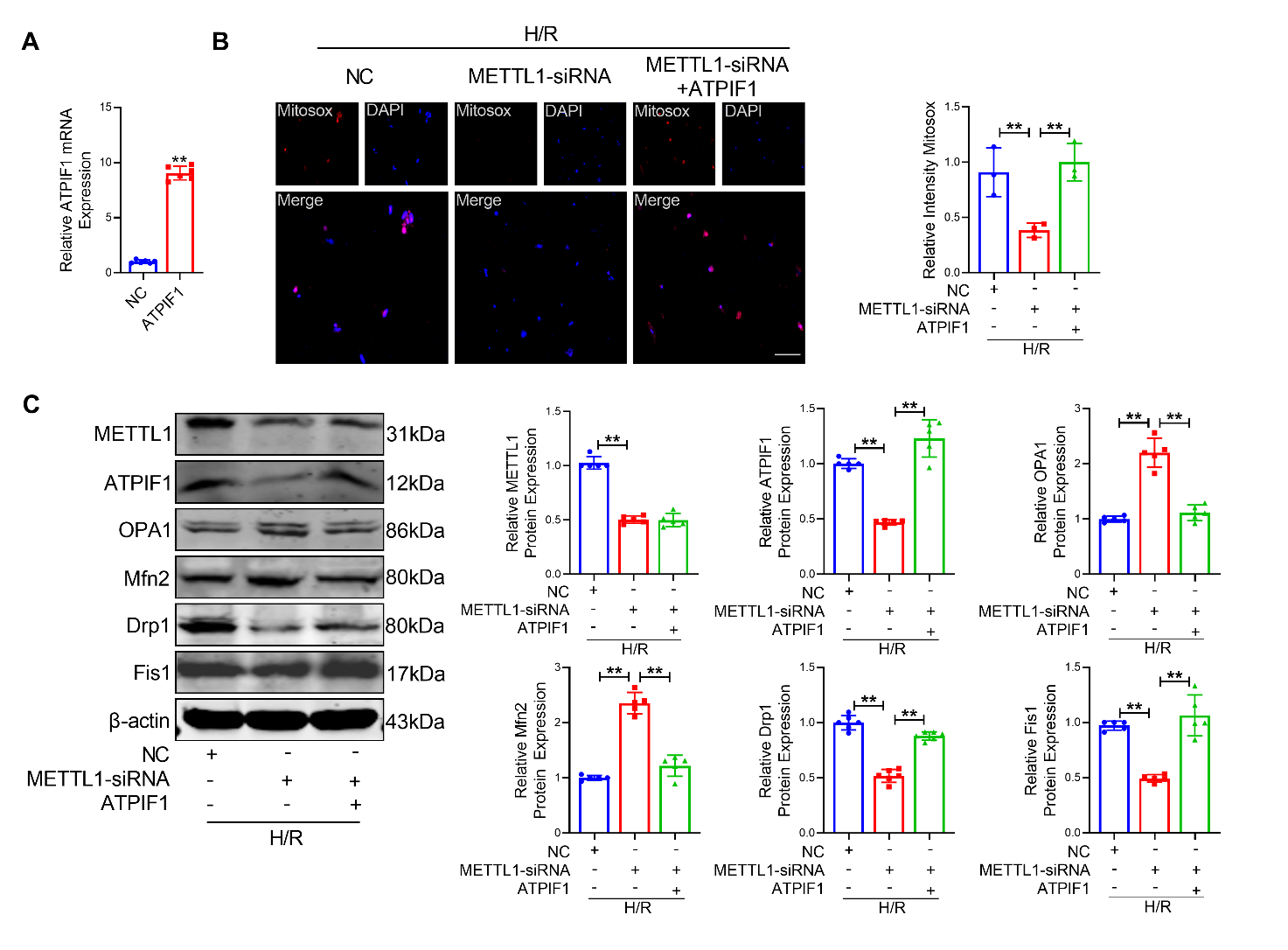
**

**Fig. S7 ATPIF1 blocks the regulation of METTL1 on mitochondrial dysfunction. (A)** qRT-PCR analyzed the mRNA expression of ATPIF1 in CMs with transfection ATPIF1 plasmid. n=6. **(B)** Mitosox staining in CMs was used to detect mitochondrial ROS. Scale bar: 50μm. n=3. **(C)** The protein levels of OPA1, Mfn2, Drp1, Fis1, and ATPIF1 in CMs. n=5. Values represent the mean ± SD. ^*^*P* < 0.05, ^**^*P* < 0.01.

**Figure S8**


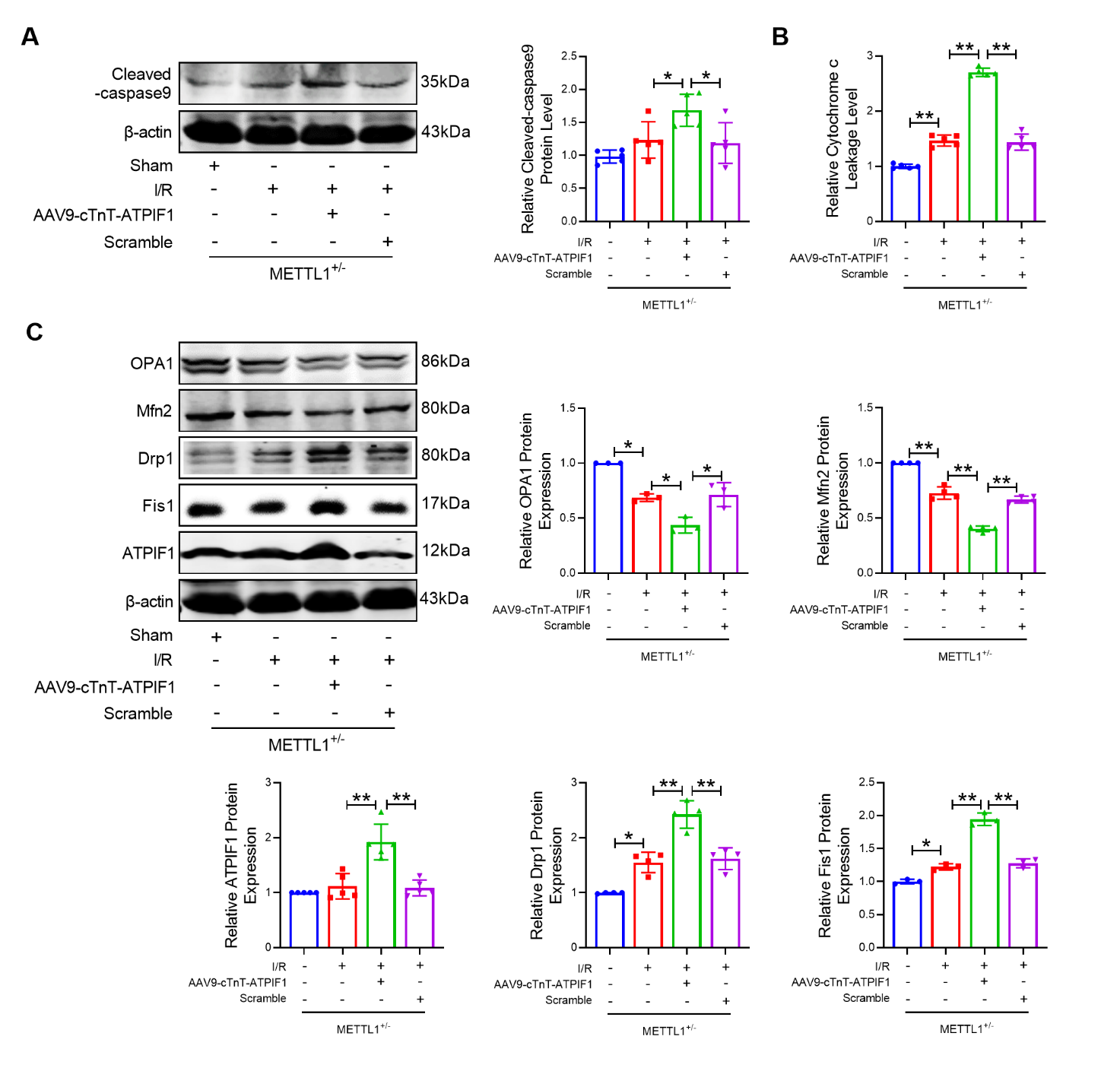


**Fig. S8 ATPIF1 reversed the therapeutic effect of knockdown METTL1.** **(A)** The protein levels of Cleaved-caspase9 in METTL1^+/-^ mice and METTL1^+/-^ mice with AAV9-cTnT-ATPIF1 after I/R injury by western blot. n=5. **(B)** The leakage level of cytochrome c. n=5. **(C)** The protein levels of OPA1, Mfn2, Drp1, Fis1, and ATPIF1. n=3. Values represent the mean ± SD. ^*^*P* < 0.05, ^**^*P* < 0.01.

**Materials and Methods**

**Echocardiography**

Echocardiography was performed using the echocardiography system Vevo 2100 (Vevo 2100; Visual Sonics, Canada). The probe frequency was 30 MHz to evaluate left ventricular function. At 24 hours after surgery, all mice were anesthetized with 1.5-2% isoflurane throughout the procedure for non-invasive examination (heart rates were maintained >450 beats/min). A 2-D image was obtained of the mouse papillary muscle. The EF and FS were measured on the M-shaped curve, and statistical analysis was performed based on the average of the three cardiac cycles.

**Infarct size detection**

The cardiac tissues were removed and washed with phosphate-buffered solution. After freezing, the tissues were cut into four pieces and stained with a TTC (Solarbio, G3005, China) dyeing solution for 8 minutes. The infarcted area (white in color) was identified via Image analysis software.

**Hematoxylin-eosin and immunohistochemical staining of the heart tissues**

After 4% paraformaldehyde was used to fix the heart tissue, the heart tissue was embedded in paraffin to make 5 μm section. The sections after dewaxing were stained with hematoxylin-eosin (H&E). To conduct IHC analysis, cardiac tissue sections (5 μm) were exposed to METTL1 or ATPIF1 at 4 ℃ for 24 hours, followed by treatment with anti-mouse or anti-rabbit HRP reagent at room temperature for 1 hour. The sections were rinsed in PBS and counterstained with hematoxylin. Images of these stained sections were examined using a microscope (Olympus Optics, Tokyo, Japan) and photographed.

**ATP detection**

The tissue, cells, are into the enzyme-free 1.5 mL EP tube, adding ATP detection lysis solution of about 100 μL-200 μL, so that the tissue or cell is completely lysis, lysis after centrifugation 4 ℃, 12000r, 5 minutes, take the supernatant for use. 20 μL standard and tissue samples were added to the hole where ATP detection working fluid (Beyotime, S0026, China) was added. The values of RLU were detected in a multifunctional microplate reader with a chemiluminescence detection function.

**Flow cytometric analysis of apoptosis**

Apoptosis was quantitatively assessed by flow cytometry using Annexin V-FITC/PI dual staining. Data acquisition was performed on a [BD FACSCanto/Beckman Coulter CytoFLEX] system, with ≥10,000 events analyzed per sample. Compensation controls ensured minimal spectral overlap between fluorescence channels.

**Analysis of Oxygen Consumption Rate**

According to the manufacturer’s recommended protocol, OCR was analyzed by the Seahorse XFe24 analyzer (Agilent Technologies, California, USA). In short, the cells were added to the hippocampal cell culture microplate before measurement. After transfection and H/R treatment, DMEM was removed and replaced with Seahorse XF DMEM culture. Drugs: oligomycin (1.5 μM), rotenone/antimycin A (0.5 μM), and FCCP (4 μM) were added in turn to monitor OCR in real time. All reagents are from Agilent Technologies. Finally, the cells in the culture plate were digested with EDTA and the cells were counted. The results were analyzed by Seahorse Wave Desktop 2.6.3.5 (Agilent).

**MeRIP-tRNA sequencing**

Three hearts of WT mice suffering cardiac I/R surgery and three hearts of METTL1+/- mice suffering cardiac I/R surgery were prepared. After sampling, the residual blood in the heart was squeezed out, placed in an enzyme-free EP tube, frozen in liquid nitrogen, and then performed MeRIP-tRNA-seq (Shanghai Yunxu Biotechnology Co., Ltd.). Briefly described as follows: fragmented RNA and m7G antibody were incubated at 4 ℃ in IPP buffer for 2 hours. The reaction mixture was further immunoprecipitated with protein A magnetic beads for 2 hours at 4 degrees. The bound RNA on the beads is then eluted with a free m7G adenosine analog. The eluted RNA was screened for fractions less than 200 nt in length using the MirVana Isolation Kit. This portion of RNA was deaminoacylated by incubating 0.1 M Tris-HCl, pH 9.0, and 1 mM EDTA at 37 ℃ for 30 minutes. tRNA-sequencing libraries were constructed using the TruSeq Small RNA Preparation Kit. All libraries were screened for 170-210 bp fragments and sequenced on Illumina NovaSeq sequencer.

**tRNA sequencing**

In brief, the MirVana Isolation Kit was used to enrich small RNA with a length of less than 200 nt from total RNA. The enriched small RNA was treated at 37 ℃ for 30 minutes in 0.1 M Tris buffer (pH 9.0) containing 1 mM EDTA. According to the GenSeq Small RNA Library Prep Kit instructions, a small RNA library was constructed with the treated samples. Fragment screening was used to enrich the library within the tRNA length range, followed by sequencing on an Illumina NovaSeq sequencer.

**Ribosome profiling sequencing**

We transfected METTL1-siRNA and NC-siRNA into primary cardiomyocytes treated with H/R, respectively, and performed ribo-seq sequencing on three samples in each group (Shanghai Yunxu Biotechnology Co., Ltd.). In brief, after being treated with cycloheximide, the cells were lysed using lysis buffer and subsequently digested using nuclease. Size exclusion columns were used to separate the digested samples. By using polyacrylamide gel electrophoresis to select the ribosome-protected RNA fragments, and the rRNA was extracted using a rRNA removal reagent. The purified RNA ends were repaired with a 3 ' adaptor and converted into cDNA by reverse transcription. Polyacrylamide gel electrophoresis was used to purify the cDNA, which was then amplified using PCR after cyclization. The amplified library was purified and sequenced using the NovaSeq sequencer.

**Creatine Kinase MB Isoenzyme**

According to the product instructions, the CK-MB test kit (Nanjing Jiancheng Biotechnology, H197-1-2, China) was used to assess the myocardial CK-MB.

**Cardiac Troponin T**

According to the product instructions, the cTn-T test kit (Nanjing Jiancheng Biotechnology, H149-4-2, China) was used to assess the myocardial CK-MB.

**Quantification of Cytochrome c Release by ELISA**

Resuspend the cells or tissue in pre-chilled mitochondrial isolation buffer (containing 0.25 M sucrose, 1 mM EDTA, 10 mM Tris-HCl, pH 7.4) and homogenize on ice. Centrifuge the homogenate at 800×g for 10 min at 4°C to remove unbroken cells and nuclei. Collect the supernatant and centrifuge again at 10,000×g for 15 min at 4°C; the resulting supernatant represents the cytosolic fraction. Measure cytochrome c levels in the cytosolic fraction using ELISA kit.

**Reverse transcription and quantitative real-time PCR**

According to the manufacturer’s protocol, total RNA was extracted with Trizol. Following that, they were reverse-transcribed into cDNA. qRT-PCR was used to quantify the expression of target gene mRNA using SYBR Green Master Mix. And the β-actin was used as endogenous control. The sequences of primer pairs used in this study are presented in Supplemental Table 2.

**Assessment of the mPTP opening in CMs**

Calcein-AM was used to determine the mPTP opening. The cells were loaded with calcein-AM and cytoplasmic calcein fluorescence quencher (CoCl_2_) at 37 ℃ for 30 minutes. Hoechst 33342 staining was used to stain the nucleus. Images were collected under a confocal laser scanning microscope.

**JC-1 staining**

JC-1 fluorescent mitochondrial imaging was used to identify the mitochondrial membrane potential. According to the product instructions, after 20 minutes of JC-1 incubation at 37 °C, the cells were rinsed using JC-1 buffer. Next, it was placed under the laser confocal microscope to observe and obtain the picture.

**TUNEL staining**

The TUNEL fluorescence kit (Roche, USA) detects the DNA fragmentation of single cells. Penetrate heart tissue or CM with 0.3% Triton X-100, then incubate with TUNEL reagent at 37 ℃ for 1 hour. Then it was placed under the laser confocal microscope to observe and obtain the picture

**Mitosox^TM^ staining**

According to the instructions, the Mitosox^TM^ working solution = 1: 1 was added to a 24-well plate with 250 μL of two solutions, gently mixed, placed in an incubator at 37 ℃, and incubated in the dark for 15 minutes. After the incubation, the staining solution was washed off, washed three times with DPBS, 5 minutes each time, sealed with an anti-fluorescence quencher containing DAPI, and dried with filter paper. After the liquid of the rounded sheet is sucked, it was placed under the laser confocal microscope in time to observe and obtain the picture.

**Wheat germ agglutinin**

The cross-sectional area of left ventricular (LV) cardiomyocytes was visualized by staining with fluorescein-conjugated wheat germ agglutinin and assessed by calculating the cross-sectional area of individual cardiomyocyte using ImageJ.

**Lactate dehydrogenase**

According to the product instructions, the LDH test kit (Nanjing Jiancheng Biotechnology, A020-2, China) was used to assess the myocardial LDH activity. LDH release is expressed in units per liter (U/L).

**Supplementary Table 1**

**Antibodies used in this study.**

| Antibodies | source | identifier |
| --- | --- | --- |
| METTL1 | Proteintech | Cat#14994-1-AP |
| WDR4 | SignalWay Antibody | Cat#42846 |
| cleaved-caspase9 | Proteintech | Cat# 10380-1-AP |
| cleaved-caspase3 | Proteintech | Cat No. 25128-1-AP |
| Bax | Proteintech | Cat# 50599-2-Ig |
| Bcl2 | ABclonal | Cat#A0208 |
| OPA1 | Cell Signaling Technology | Cat# 80471 |
| Mfn2 | Snata | Cat#sc-100560 |
| Drp1 | Snata | Cat# sc-271583 |
| Fis1 | ABclonal | Cat#A19666 |
| ATP5G3 | Origo | Cat#ARG42716 |
| N^7^-methylguanosine (m^7^G) antibody | MBL International | Cat# RN017M |
| anti-puromycin antibody | Millipore | Cat# MABE343 |
| β-actin | Affinity | Cat#T0022 |
| XIAP | Proteintech | Cat # 23453-1-AP |

**Supplementary Table 2**

**Primers used for the qRT-PCR assay**

| NO. | sequence (5’ - 3’) |
| --- | --- |
| METTL1-F | GAACATCGCCTGTCTCCGAA |
| METTL1-R | TCGCTTAAAGTGTGGGTCCG |
| WDRE-F | TCTCCAAGTCTGGCCGCTAT |
| WDR4-R | CGCACCACCATCCTGACACT |
| ATPIF1-F | GGTGTCTGGGGTATGAAGGTC |
| ATPIF1-R | CCTTTTCTCGTTTTCCGAAGGC |
| ANP-F | CTCCGATAGATCTGCCCTCTTGAA |
| ANP-R | GGTACCGGAAGCTGTTCGAGCCTA |
| BNP-F | TTGGGCAGAAGATAGACCGGAT |
| BNP-R | GGTCTTCCTAAAACAACCTCA |
| β-MHC-F | ATGTGCCGGACCTTGGAA |
| β-MHC-R | CCTCGGGTTAGCTGAGAGATCA |
| ATP5G3-F | GTGTGTCAGCTGATCCGAAG |
| ATP5G3-R | AGGCAAATCCCAGGATAGCA |
| SLC25A3-F | CTGGTGCACGATGGCCTG |
| SLC25A3-R | AACCAAGCTGTATGTGT |
